# Mapping the epithelial–immune cell interactome upon infection in the gut and the upper airways

**DOI:** 10.1038/s41540-022-00224-x

**Published:** 2022-05-02

**Authors:** Martina Poletti, Agatha Treveil, Luca Csabai, Leila Gul, Dezso Modos, Matthew Madgwick, Marton Olbei, Balazs Bohar, Alberto Valdeolivas, Denes Turei, Bram Verstockt, Sergio Triana, Theodore Alexandrov, Julio Saez-Rodriguez, Megan L. Stanifer, Steeve Boulant, Tamas Korcsmaros

**Affiliations:** 1 Earlham Institute, Norwich Research Park, Norwich, UK; 2 Quadram Institute Bioscience, Norwich Research Park, Norwich, UK; 3 Department of Genetics, Eotvos Lorand University, Budapest, Hungary; 4 Faculty of Medicine, Heidelberg University, Heidelberg, Germany; 5 Institute for Computational Biomedicine, Heidelberg University Hospital, Heidelberg, Germany; 6 Department of Gastroenterology and Hepatology, University Hospitals Leuven, KU Leuven, Leuven, Belgium; 7 Department of Chronic Diseases and Metabolism, Translational Research in GI disorders, KU Leuven, Leuven, Belgium; 8 Structural and Computational Biology Unit, European Molecular Biology Laboratory, Heidelberg, Germany; 9 Collaboration for joint PhD degree between EMBL and Heidelberg University, Faculty of Biosciences, Heidelberg, Germany; 10 Skaggs School of Pharmacy and Pharmaceutical Sciences, University of California San Diego, La Jolla, CA USA; 11 Molecular Medicine Partnership Unit (MMPU), European Molecular Biology Laboratory, Heidelberg, Germany; 12 Department of Infectious Diseases, Heidelberg University Hospital Heidelberg, Heidelberg, Germany; 13 Department of Metabolism, Digestion and Reproduction, Imperial College London, London, UK

**Keywords:** Computational biology and bioinformatics, SARS-CoV-2, Multicellular systems, Gastroenterology

## Abstract

Increasing evidence points towards the key role of the epithelium in the systemic and over-activated immune response to viral infection, including SARS-CoV-2 infection. Yet, how viral infection alters epithelial–immune cell interactions regulating inflammatory responses, is not well known. Available experimental approaches are insufficient to properly analyse this complex system, and computational predictions and targeted data integration are needed as an alternative approach. In this work, we propose an integrated computational biology framework that models how infection alters intracellular signalling of epithelial cells and how this change impacts the systemic immune response through modified interactions between epithelial cells and local immune cell populations. As a proof-of-concept, we focused on the role of intestinal and upper-airway epithelial infection. To characterise the modified epithelial–immune interactome, we integrated intra- and intercellular networks with single-cell RNA-seq data from SARS-CoV-2 infected human ileal and colonic organoids as well as from infected airway ciliated epithelial cells. This integrated methodology has proven useful to point out specific epithelial–immune interactions driving inflammation during disease response, and propose relevant molecular targets to guide focused experimental analysis.

## Introduction

Specialised epithelial cells lining the surface of the mammalian gastrointestinal tract form the primary line of defense against external stimuli, working in cohort with resident immune cells to maintain homeostasis and defend the body from infections. Although the role of both the epithelium and the immune system during infection have been assessed in previous studies, these components have often been investigated separately. While this knowledge has been instrumental in advancing medical research, the recent COVID-19 pandemic has pointed out the need for large-scale, integrative models to address key questions that cannot yet be solved with available experimental models. One example is: what is the role of an infected cell in inducing systemic inflammatory responses by communicating to resident immune cells? To address this critical question, existing yet often disconnected datasets and computational approaches can be leveraged to develop a complex but easily interpretable map of how viral molecules are able affect different cell populations in the gut, and how infected cells can in turn modulate local and systemic immune and inflammatory responses.

The recent COVID-19 pandemic is caused by infection with the severe acute respiratory syndrome coronavirus 2 (SARS-CoV-2). While SARS-CoV-2 mainly targets the lung and upper airways^1–3^, other organs can be infected too, including the heart, kidney, brain, and the intestine^4^. In addition to directly infecting key organs, the main hurdle of SARS-CoV-2 infection is the excessive inflammatory response mediated by both the innate and adaptive immune systems^1,5^. The overactivated inflammatory response, also known as cytokine release syndrome (CRS) or cytokine storm, is the result of high levels of circulating cytokines and chemokines, and it is thought to be responsible for the severe COVID-19 symptoms some patients experience^6^. Yet, there is no clear understanding of which particular inflammatory pathways and cell types are responsible for driving this process, and whether some organs are more important than others in the initiation and maintenance of this syndrome^7^. The causal role of SARS-CoV-2 on intestinal damage and the role of the small intestine in contributing to CRS was recently highlighted^8,9^.

Human intestinal organoids have been used as a tool to study SARS-CoV-2 infection in the gut and the inflammatory responses of specific intestinal epithelial cell types^10–13^. These studies provided evidence that SARS-CoV-2 is able to infect and actively replicate in human intestinal cells, in particular in enterocytes^10,13^. These studies also revealed that, contrary to the limited type I and type III interferon (IFN) immune response observed in the lungs^14,15^, the response to SARS-CoV-2 infection in the gut is characterised by a cell-type specific inflammatory response that is important in the development of systemic reactions^11^. Examination of human intestinal samples has also shown that infection of gut epithelial cells results in the activation of local immune populations^16^. Yet, the exact effects of viral infection in the gut and the role of epithelial cell–immune cell interaction in mediating the inflammatory response of the body are not known. This information could ultimately aid the development of treatments and strategies to optimize the level and type of immune response as we would understand better the viral strategies that dysregulate our immune system. Due to the lack of adequate and complex experimental systems, to the best of our knowledge, no study has been carried out so far to analyse epithelial–immune crosstalk in the gastrointestinal tract upon SARS-CoV-2 infection.

Here, we introduce a computational framework to map epithelial–immune interactions, improve our understanding in the gut or in other organs by interpreting existing data better, and importantly, provide a short list of key molecules for targeted experimental validations. To achieve this, we integrated two previously developed intracellular modelling tools (ViralLink and CARNIVAL) with intercellular network approaches (from OmniPath)^17–19^. We present two proof of concept studies on intestinal organoids and upper airways ciliated cells. We used available SARS-CoV-2–human mRNA/protein–protein interaction predictions to model the effect of viral infection on intracellular signalling networks in host intestinal and ciliated cells, and applied published single-cell datasets to create cell-type, organ and context-specific epithelial–immune interaction maps. We demonstrated the importance and usefulness of this map with integrated analyses, which provided an improved understanding of the effect of viral infection on ileal, colonic and airway epithelial cells, and the role of epithelial–immune cell crosstalk during SARS-CoV-2 infection. Ultimately, this framework may help to find key intercellular inflammatory pathways involved in these crosstalks that could pave the way for potential successful strategies against the cytokine release syndrome associated-symptoms observed in severe cases of COVID-19. Importantly, the presented integrated framework will allow investigating other infections and conditions for which our analytical toolkits can be repurposed.

## Results

### Reconstructing the intestinal epithelial–immune interactome

**Fig. 1:**
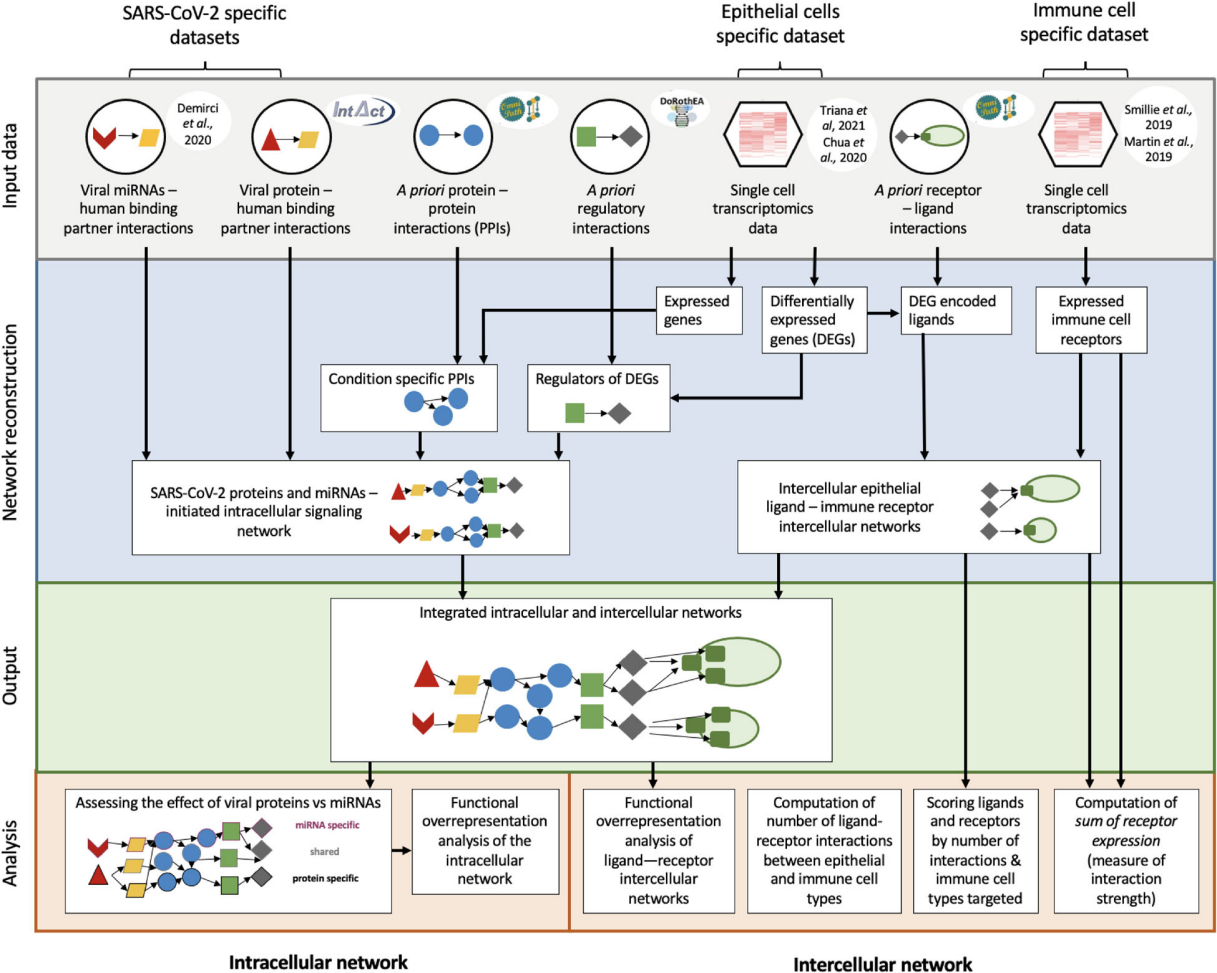
Integrated workflow to analyse the intracellular and intercellular effect of SARS-CoV-2 in the gut. Schematic workflow illustrating the different analytical steps used to construct the intracellular and intercellular signalling networks between epithelial cells in SARS-CoV-2 infected intestinal organoids (ileal and colonic organoids, 24 h infection) or moderate COVID-19 upper airway ciliated epithelial and immune cell types.

In our previous work, we identified a subpopulation of enterocytes as the prime target of SARS-CoV-2 (BavPat1/2020 strain), with directly infected cells showing a high pro-inflammatory response and little to no interferon-mediated response as the result of a SARS-CoV-2-mediated inhibition of interferon signalling^13^. These findings highlighted the key role of the gut as a pro-inflammatory reservoir, which primes for further investigation to be able to fully understand SARS-CoV-2 pathogenesis. Building on this study, we created an integrated bioinformatics framework that enables the investigation of the infected epithelial cell–immune cell crosstalk in a cell-type specific manner (Fig. 1). To do so, we exploited a priori knowledge on ligand–receptor interactions [18] to construct intercellular networks connecting epithelial cells and resident immune cells using our previously generated single cell RNA-seq epithelial cell dataset of ileal and colonic organoids infected with SARS-CoV-2 (BavPat1/2020 variant)^13^ and a separate lamina propria immune cell one^20,21^.

First, we looked at the epithelial cell population with the highest number of ligands among the differentially expressed genes upon infection to assess which epithelial cell type could drive the response to SARS-CoV-2 infection. Our previous findings^13^ had highlighted immature enterocytes (originally known as “immature enterocytes 2”, an enterocyte subpopulation characterised by MMP7+, MUC1+, CXCL1+) as the epithelial population characterised by the highest number of differentially expressed genes upon infection (Supplementary Fig. 1). In accordance with this, we found that this population was also characterised by the highest number of differentially expressed ligands. Hence, differentially expressed ligands of colonic and ileal immature enterocytes were used to build epithelial–immune intercellular networks by connecting ligands to their binding receptors on immune cells (Fig. 1 and Methods).

**Fig. 2:**
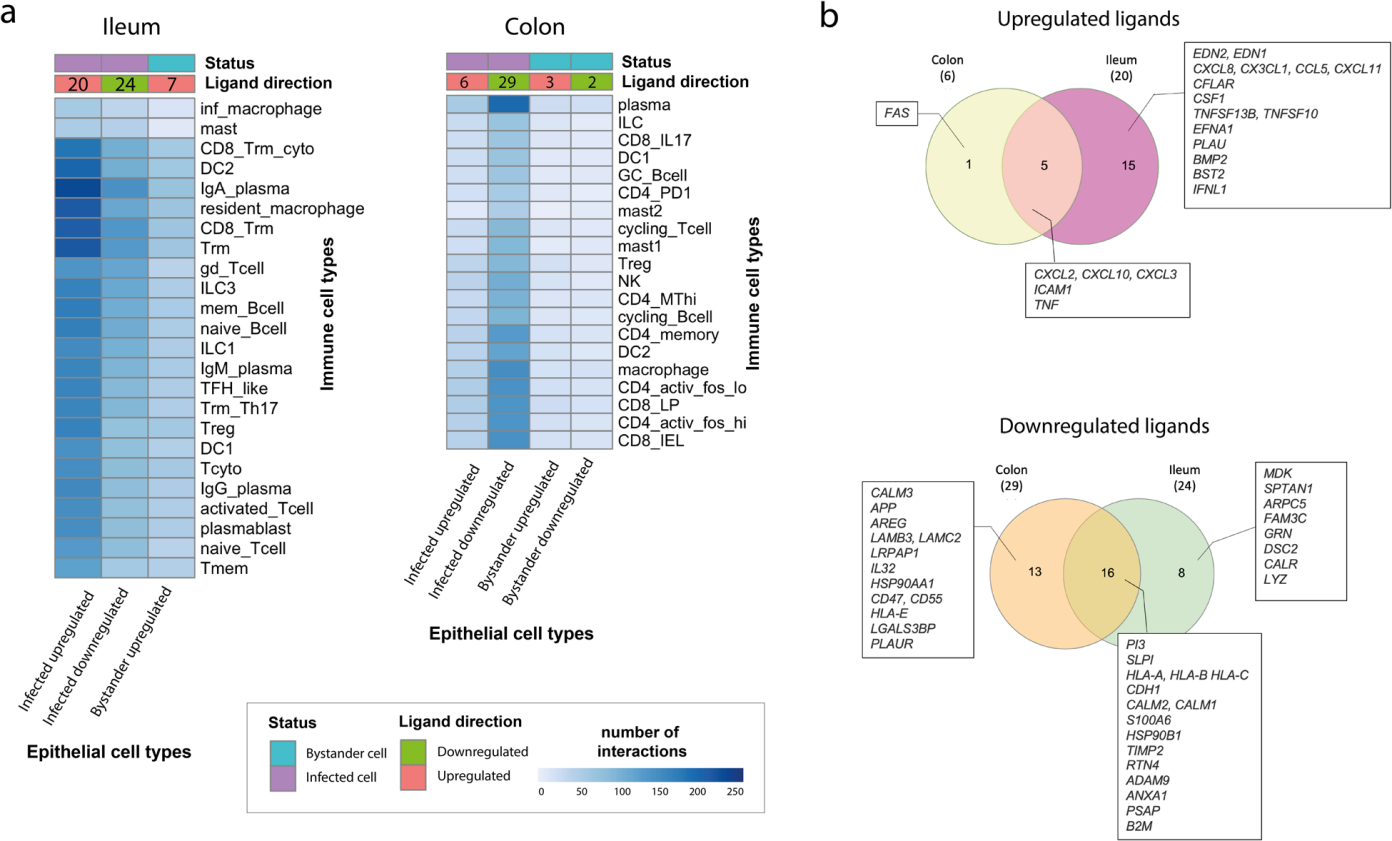
Differentially expressed ligands driving upregulated and downregulated intercellular interactions between colonic and ileal infected immature enterocytes and resident immune cells upon infection in the colon and ileum. **a** Heatmap showing the number of interactions between immature enterocytes and resident immune cells. Interactions driven by upregulated and downregulated ligands (ligand direction) are shown separately for infected and bystander cells (status), and for ileum and colonic organoids. The intensity of the colour indicates the number of interactions with the immune cell types whose receptor is targeted by the epithelial cells ligands. The numbers on the ligand direction row refer to the number of upregulated or downregulated ligands driving the indicated interactions with immune cells for the different groups/conditions. Abbreviations: *Ileum:* inf_macrophage infected macrophage, mast mast cell, CD8_Trm_cyto Resident memory cytotoxic T cell, DC2 dendritic cell 2, Trm Tissue-resident memory T cell, gd_Tcell Gamma delta (γδ) T cells, ILC Innate lymphoid cell, mem_Bcell memory B cell, naive_Bcell naive B cell, TFH_like T follicular helper cells, Trm_Th17 Tissue-resident memory Th17 cells, Treg Regulatory T cell, Tcyto Cytotoxic T cell, Tmem Memory T cells. *Colon*: ILC Innate lymphoid cell, CD8_IL17 IL-17+ CD8+ T cells, DC dendritic cells, GC_Bcell Germinal center B cells, CD4_PD1 mast mast cell, Treg Regulatory T cell, NK Natural Killer cell, CD4_MThi high mitochondrial CD4+ T cell, CD4_memory CD4+ Memory T cell, CD4_activ_fos_high activated CD4+ T cells (high/low c-fos), CD8_LP CD8+ lymphocyte-predominant cells, CD8_IEL CD8+ intraepithelial lymphocytes. **b** Venn diagrams showing the number of ligands of the infected immature enterocytes–immune cells intercellular network that are unique or shared between the ileum and colon. Upregulated and downregulated ligands are shown separately. The full list of ligands is available as Table 1.

To identify the main epithelial and immune cell types involved in intercellular crosstalk, the putative number of ligand–receptor interactions between each epithelial–immune cell type pair was computed. Specifically, all possible interactions between each set of up or downregulated epithelial ligands and each of the receptors expressed by the specific immune cell type (from^20^ and^21^) were identified (Fig. 1 and Methods). While both bystander and infected cell populations were affected by viral infection, directly infected intestinal cell populations had a higher number of predicted interactions with immune cells compared to bystander cell populations in both colon and ileum, supporting a role for direct viral infection in altering intercellular signalling in the gut (Fig. 2a). In the colon, the higher number of epithelial–immune interactions was identified between downregulated ligands of infected immature enterocytes and plasma cells, as well as CD4+/CD8+ T cells, macrophages and dendritic cells (DCs) to a lesser extent (Fig. 2a). Conversely, in the ileum, the highest number of interactions was identified between upregulated ligands of infected immature enterocytes and IgA plasma cells, T resident memory (Trm) cells, dendritic cells and resident macrophages (Fig. 2a). Notably, the higher number of interactions in the ileum was not a result of a higher number of upregulated ligands (20), as this was similar to the number of downregulated ones (24) (Fig. 2a, b). Instead, the higher number of interactions was driven by upregulated ligands binding to multiple receptors on each immune cell targeted. A more detailed explanation can be found in the Supplementary Results.

**Table 1:** Ligands in ligand–receptor interactions in the colon and ileum.

Tissue	Direction	Number	Ligands
Colon only	Upregulated	1	FAS
Colon only	Downregulated	13	CALM3, APP, AREG, LAMB3, LRPAP1, IL32, CD47, HSP90AA1, CD55, LAMC2, HLA-E, LGALS3BP, PLAUR
Ileum only	Upregulated	15	EDN2, CXCL8, CX3CL1, CFLAR, CSF1, EDN1, TNFSF13B, TNFSF10, EFNA1, PLAU, CCL5, CXCL11, BMP2, BST2, IFNL1
Ileum only	Downregulated	8	MDK, SPTAN1, ARPC5, FAM3C, GRN, DSC2, CALR, LYZ
Colon and ileum	Upregulated	5	CXCL2, CXCL10, ICAM1, CXCL3, TNF
Colon and ileum	Downregulated	16	PI3, SLPI, HLA-A, CDH1, CALM2, S100A6, HSP90B1, TIMP2, RTN4, ADAM9, HLA-B, HLA-C, ANXA1, CALM1, PSAP, B2M

Table listing the number of ligands of infected immature enterocytes–immune cells intercellular network that are unique or shared between the ileum and colon.

With this integrated network reconstruction, we have shown the value of our framework in enabling the study of mechanistic details of the effect of SARS-CoV-2, or other viruses, on the human immune system.

### The infected epithelial signalling network drives the epithelial–immune interactome

To further understand how SARS-CoV-2 infection drives altered ligand expression in infected intestinal epithelial cells, we used our integrated network model to reconstruct the altered intracellular signalling in directly infected immature enterocytes population driven by SARS-CoV-2. Within our framework, two separate bioinformatics tools, ViralLink and CARNIVAL, were used to construct an intracellular causal network linking perturbed human proteins interacting with SARS-CoV-2 viral proteins or miRNAs to activated transcription factors (TFs) regulating the differentially expressed ligands upon infection, through altered intracellular protein-protein signalling cascades (Fig. 1). By integrating tissue-specific epithelial data, we have constructed two separate causal networks for infected immature enterocytes of the ileum and colon, thus enabling us to distinguish tissue specific differences in infection response (Fig. 1). Detailed analysis of the colon and ileal intracellular network features can be found in the Supplementary Results. Furthermore, using multiple complementary methods of network analysis, we have highlighted the most likely signalling pathways affected upon infection. Finally, by integrating a priori information on SARS-CoV-2 miRNAs/proteins - human protein interactions, we have built separate sublayers of the networks representing altered signalling stemming from upstream perturbations caused by SARS-CoV-2 miRNAs, proteins or both. These networks allowed us to assess the contribution of each of these viral factors in altering the intracellular signalling cascade (Fig. 1 and Methods).

**Fig. 3:**
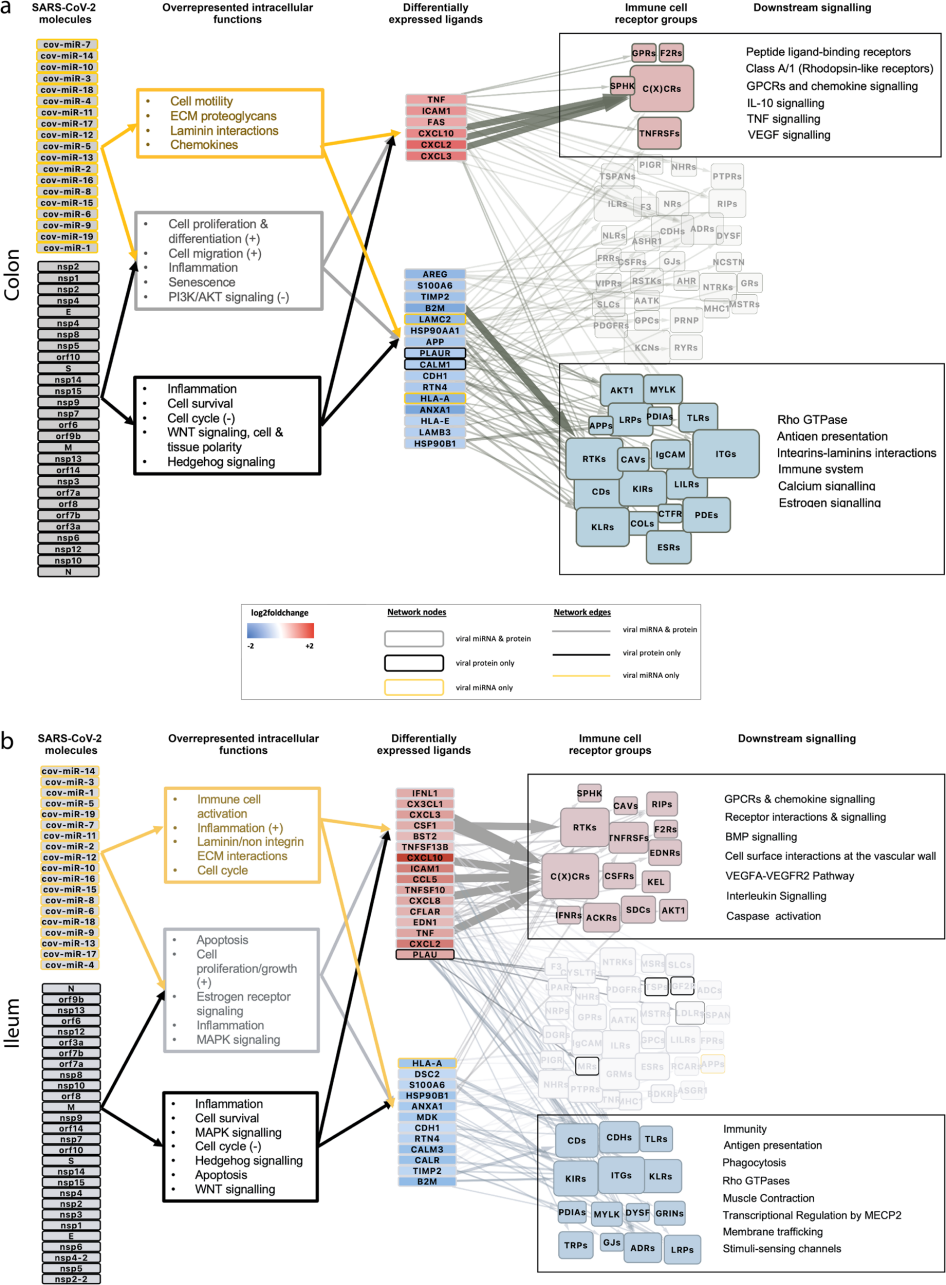
Overview of intracellular and intercellular signalling of colonic and ileal infected immature enterocytes upon SARS-CoV-2 infection. **a, b** Overview of intracellular and intercellular signalling upon SARS-CoV-2 infection in colonic (**a**) and ileal (**b**) infected immature enterocytes and immune cell populations. From left to right: signalling cascade going from SARS-CoV-2 molecules (proteins or miRNAs) to differentially expressed ligands on immature enterocytes and binding receptor groups on immune cells. Intracellular network: SARS-COV-2 molecules are grouped separately if they are viral proteins (bottom) or miRNAs (top). Differentially expressed ligands for which no upstream signalling was identified, but downstream intercellular connections were predicted are excluded from this figure. Differentially expressed ligands are grouped based on the direction of regulation, which is indicated with blue when downregulated (bottom) and red when upregulated (top) when comparing SARS-CoV-2 infected vs uninfected conditions. Colours of the nodes and of the functional analysis indicate if the original network was a miRNA only (yellow), viral protein only (black) or both viral protein and miRNA (grey). Functional overrepresentation analysis was carried out for the “PPI layer” of the intracellular network which includes human binding proteins, intermediary signalling proteins and TFs (adj *p* value < 0.05, *n* > 3). Intercellular network: Size of the receptor node represents the sum of receptors within the group targeted by each incoming ligand. Functional analysis is indicated for ligand–receptor groups. Receptor groups layout is based on whether they contributed to the functional analysis of upregulated interactions (red) or downregulated interactions (blue). Receptor groups not contributing to any functions are indicated in light grey.

Functional analysis of the tissue-specific intracellular networks is useful to understand how SARS-CoV-2 infection in immature enterocytes affects their function through the modulation of intracellular signalling, and whether any differences in response exist between colon and ileum. Here, a functional overrepresentation analysis (Gene Ontology (GO) and Reactome) of the protein-protein interaction (“PPI”) layer^22–25^ of each intracellular causal sub-network (stemming from viral proteins, miRNAs, or both) was integrated in our framework to assess the contributions of SARS-CoV-2 miRNAs or proteins to the changes observed^22–25^ (Fig. 1 and Methods). Functional analysis of the PPI layer of the intracellular networks built with ViralLink revealed an overrepresentation of pathways related to inflammation and chemotaxis (Nuclear Factor kappa-light-chain-enhancer of activated B cells (NF-kB) signalling, interleukin signalling, chemokine signalling) in both ileum and colon (Fig. 3). Additionally, we found the overrepresentation of functions related to interferon (IFN) signalling and Mitogen-Activated Protein Kinase (MAPK) signalling being overrepresented uniquely in the ileum in both viral protein and miRNA intracellular networks (Fig. 3b). An overrepresentation of laminin-driven interaction pathways, which we observed uniquely for viral miRNA intracellular network in both ileum and colon, could be indicative of an increased recruitment and adhesion of immune cells following infection (Fig. 3). Furthermore, an overrepresentation of pathways related to negative regulation of apoptosis, cell cycle, cell proliferation and growth was found in both ileal and colonic networks, suggesting an effect of SARS-CoV-2 on epithelial cell tissue renewal (Fig. 3). Interestingly, an overrepresentation of WNT signalling pathway, which is key for stem cell renewal, was found uniquely in the viral protein sub-network, in both tissues, while pathways related to the establishment of cell and tissue polarity were found uniquely in the colon, indicating an attempt for tissue healing following viral infection (Fig. 3).

Functional overrepresentation analysis of the PPI layer of the intracellular networks built with CARNIVAL confirmed similar affected functions upon infection as those found in the ViralLink networks, suggesting functional overlap between networks obtained using these two methods such as senescence or inflammation (Fig. 3 and Supplementary Figs. 3 and 4). Within the outlined framework, the choice of which tool (ViralLink or CARNIVAL) to be used to build the intracellular network for a particular study should be driven by the specific study aims and biological questions. Further information about similarities and differences between the two tools is available in the Methods and Supplementary Results.

Further analysis of these networks can help predict key transcription factors responsible for the upstream regulation of altered ligands upon infection. Here, we found that both colonic and ileal networks generated with CARNIVAL shared similar transcription factors, including ATF2/3, FOS, JUN, STAT1, and NFKB1, which were all upregulated in both tissues upon infection (Supplementary Figs. 3 and 4). These transcription factors play a role in interferon response (STAT1^26^), and inflammation (NFKB1^27^), anti-apoptosis and cell growth (ATF2/3^28^), cell proliferation and differentiation (JUN, FOS^29^), suggesting an increase in these functions upon SARS-CoV-2 infection in both colon and ileum. Interestingly, viral miRNAs were predicted to target different intracellular signalling processes between colon and ileum (miR_10,11,16,18 in the colon and miR_4,5,6,18 in the ileum). Additionally, by analysing these networks, we observed that NOTCH1 and SMAD4, seem to be central to the intracellular signalling cascade in the colon, by receiving several signals driven by viral miRNAs and viral proteins, respectively (Supplementary Fig. 3). Interestingly, both the Notch and TGF-β SMAD-dependant signalling pathways are involved in intestinal epithelial cell homeostasis, including stem cell maintenance, progenitor cell proliferation^30^ and maintenance of cell differentiation^31^, suggesting a modulation of these pathways upon infection. In the ileal network, JAK2 and CREB1, as well as SMAD2, SMAD3 and ERK2 (MAPK1) seem to play a central role in the intracellular PPI signalling driven by viral miRNAs and viral proteins, respectively, and *JAK2* and both *SMAD2* and *SMAD3* were also upregulated upon infection (Supplementary Fig. 4). These transcription factors play a key role in the regulation of immunity (JAK2, CREB1;^32,33^, cell proliferation and differentiation (MAPK1) and plasticity (SMAD2/3)^34,35^, suggesting a positive regulation of these functions uniquely in the ileum upon infection.

Reconstruction and analysis of perturbed intracellular signalling in infected enterocytes using two complementary methods highlighted key pathways through which SARS-CoV-2 affects the infected cells, and pointed out transcription factors playing a major role during SARS-CoV-2 infection response.

### Altered epithelial-derived ligands drive differential epithelial–immune crosstalk upon infection

To understand the functional impact of epithelial infection on the epithelial–immune interactome, we created intercellular networks by connecting upregulated and downregulated epithelial ligands of colonic and ileal infected immature enterocytes upon infection to their binding receptors on immune cells (Fig. 1 and Methods). Next, for each set of up and downregulated intercellular interactions, we looked at which ligands, receptors and immune cell types were involved in these intercellular interactions, assessing any potential similarities or differences between the colon and ileum (Fig. 1 and Methods). Here, we present the analysis relative to infected immature enterocytes–immune cell interaction, while the analysis relative to bystander immature enterocytes is available as Supplementary Results.

**Fig. 4:**
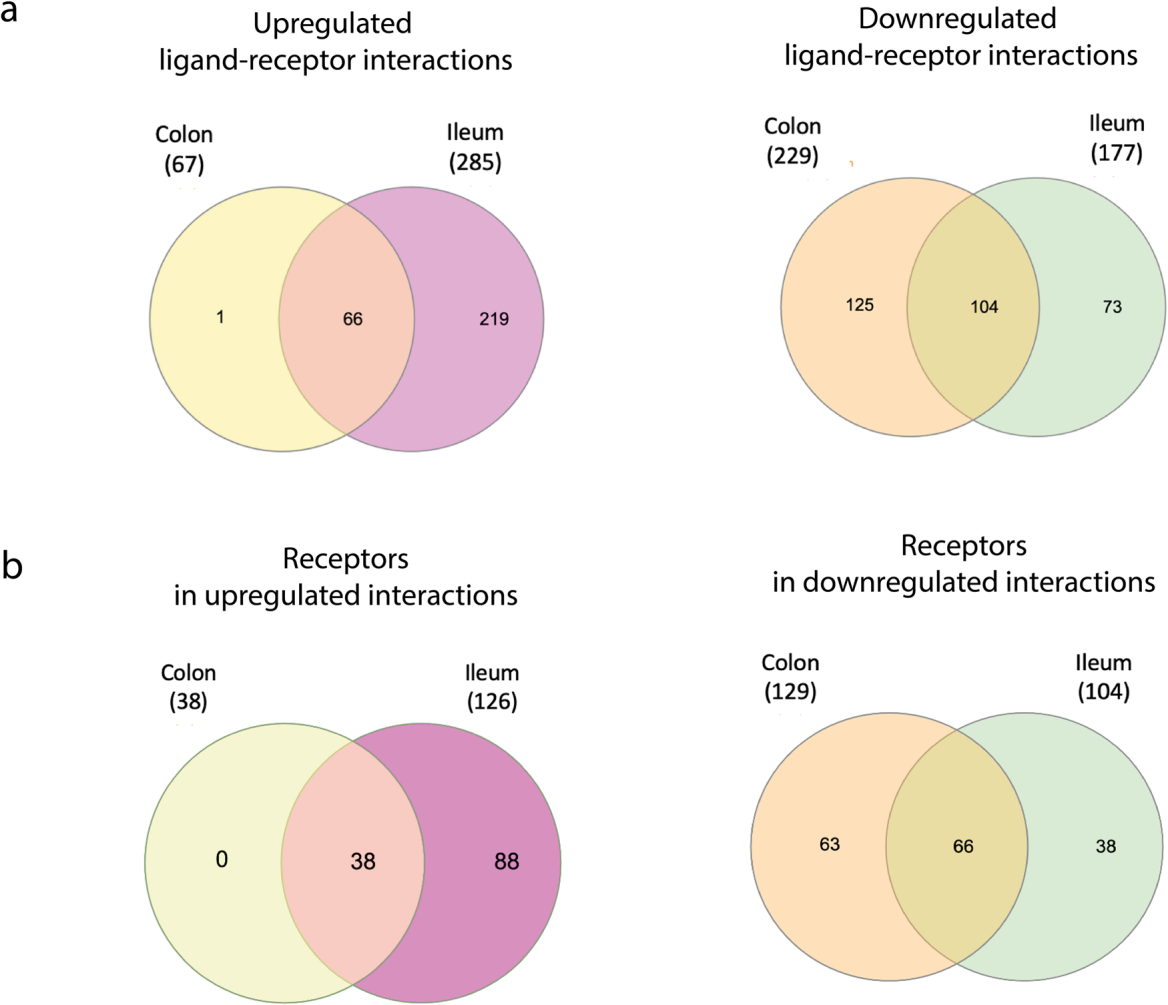
Overview of upregulated and downregulated ligand–receptor interactions and participating receptors between infected immature enterocytes and resident immune cells upon infection in the colon and ileum. **a** Venn diagrams showing the number of ligand–receptor interactions in the infected immature enterocytes–immune cells intercellular networks that are unique or shared between the ileum and colon. Intercellular interactions driven by upregulated and downregulated ligands are shown separately. The full list of ligand–receptor interactions is available as Table 3. **b** Venn diagrams showing the number of receptors in the infected immature enterocytes–immune cells intercellular networks that are unique or shared between the ileal and colonic networks. Receptors targeted by upregulated ligands and downregulated ligands are shown separately. The full list of receptors is available as Table 2.

Upregulated ligands of infected immature enterocytes upon infection as well as binding receptors on immune cells were mainly shared among colon and ileum, resulting in most epithelial–immune interactions driven by upregulated ligands being similar in both tissues (one unique to colon, 219 unique to ileum, 66 shared) (Figs. 2b, 4a, b). A full list and detailed description of differences and similarities in ligands, receptors and upregulated intercellular interactions between colon and ileum can be found as Tables 1–3 and as Supplementary Results.

**Table 2:** Receptors in ligand–receptor interactions in the colon and ileum.

Tissue	Direction	Number	Receptors
Colon only	Upregulated	0	–
Colon only	Downregulated	63	ITGA1,2,7,8,910, ITGB4,6,8, NOD2, LRP8, CD151, CD74, CD97, TGFBR1,2, SLC45A3, SLC16A, SORL1, VANGL1, FCGR2B, SIRPG, PRNP, LILRA3, PDE1A, GPC1, CFTR, PTGER1, CR1, NCSTN, AHR, COL17A1
Ileum only	Upregulated	88	MCAM, CYSLTR1/2, ACVR1/2B/L1/2 A, SLC5A11, SLC7A1, ENG, TNFRSF13B/13 C/10 C/17/10B/10D, LILRA4, CELSR3, ADGRL4, ADCY9, ACKR2/3/4, EDNRA, CCR3/6/8, CXCR1/2, EPHA10, KEL, CSF3R/2RA, EDNRB, BMPR2/R1A/R1B, SMO, AMHR2, GPR75, LPAR2, NPR3, ACKR1, IFNLR1, ST14, MRC2, CD79A, IGF2R, PTPRU, MSR1
Ileum only	Downregulated	38	TSPAN1, SCTR, KIR2DL4, KIR3DL1, GRM3, ADRB2, PTH2R, BDKRB2, GRIN2A, KLRC3, PLPP6, CRHR1, CLEC2D, GPR37, GPR37L1, ITGA2B, SCN4A, CDH2, PTPRB, AQP6, DSG2, KIR2DL1, KIR3DL3, MIP, KCNQ3, TRPC3, GRM4, SCARF1, RTN4RL1, GPC2, OPRM1
Colon and ileum	Upregulated	38	F2RL1, ITGAM/X, F3, CXCR3/4/5/6, CCR1/2/4/7/9, CX3CR1, XCR1, PTPRS, PIGR, GPR160, CELSR2, PPARG, CDH5, CD83, CDH11, SPHK1, TRADD
Colon and ileum	Downregulated	66	TLR1/2/4/7, VIPR1, TNFRSF19, PLD2, PLSCR1/4, SORT1, HFE, GP6, KLRC1/2/4, KLRG1, PDE1B/1 C, PDIA3, CD1A/B, CD3D/G, CD247, ITGAE, ITGA4/6, ITGB7, LILRB2, NGFR, GJB2, LRP5/6, FPR3, LDLR, RYR1, APLP2, CELSR1, KCNN4, KCNQ1/5, GLP2R, ESR1/2, CANX, IL2RB, ASGR1, KIR2DL2/3, PTPRA, DYSF, TRPC1

Table listing number of receptors involved ligand–receptor interactions in the infected immature enterocytes–immune cells intercellular network that are unique or shared between the ileum and colon. The direction column indicates the direction of the expression change of the epithelial ligand driving each intercellular interaction.

**Table 3:** Ligand–receptors in intercellular interactions in the colon and ileum.

Tissue	Direction	Number	Ligand–receptor interactions
Colon only	Upregulated	1	FAS _ RIPK1
Colon only	Downregulated	125	APP _ TNFRSF21, CD74, CCR5, SLC45A3, CAV1, NCSTN, FCGR2B, GPC1, LRP1, NGFR, ADRA2A
			AREG _ LTK, CSF1R, MERTK, INSR, NTRK1/2/3, ROR1/2, PDGFRB, LMTK2, FLT3, PTK7, MET, TIE1, AATK, DDR2, MST1R, AXL, TYRO3, MUSK, RET, PDGFRA, RYK
			CALM1 _ PDE1A
			CALM2 _ PDE1A
			CALM3 _ MYLK, GP6, KCNQ1/5, PDE1A/B/C, INSR, ESR1/2, AKT1
			CD47 _ SIRPG
			CD55 _ CD97, AKT1, CR1
			HLA-C _ LILRA3
			HLA-E _ KLRC1/2/4, SLC16A4, KIR2DL3/3DL2
			HSP90AA1 _ NR3C1, CFTR, TGFBR1/2, AKT1, ITGB3, AHR, NOD2, RIPK1
			IL32 _ PTGER1, MET
			LAMB3 _ CD151, COL17A1, ITGA1/2/3/4/5/6/7/8/9/10/11/V, ITGB1/3/4/5/6/7/8, AKT1, PRNP
			LAMC2 _ AKT1, ITGA1/2/3/4/5/6/7/8/9/10/11/V, ITGB1/3/4/5/6/7/8, COL17A1, PRNP, CD151
			LGALS3BP _ VANGL1, ITGB1
			LRPAP1 _ LDLR, VLDLR, SORL1, SORT1, LRP1/8
			PLAUR _ ITGB3
Ileum only	Upregulated	219	BMP2 _ AMHR2, SMO, ACVR2A/R2B/R1/RL1, BMPR1A/1B/2, ENG, CDH11
			BST2 _ LILRA4
			CFLAR _ RIPK1
			CSF1 _ PDGFRB, DDR2, LMTK2, TYRO3, RYK, MUSK, MET, CELSR3, SLC7A1, RET, ITGB3/AV, INSR, ALK, CSF1R/2RA/3R, ROR1, MSR1/T1R, NTRK1/2/3, MERTK, TIE1, AATK, AXL, ROR2, PTK7, LTK, PDGFRA, FLT3
			CCL5 _CX3CR1, CCR1/2/3/4/5/6/7/8/9, CXCR1/2/3/4/5/6/10, ACKR1/2/4, GPR75, SDC1, ADRA2A, CD4, GRM7, XCR1
			CX3CL1_CXCR1/2/3/4/5/6, CCR1/2/3/4/5/6/7/8/9/10, CX3CR1, XCR1
			CXCL10_CXCR1/2, GRM7, CCR3/6/8
			CXCL11_XCR1, CCR1/2/3/4/6/5/7/8/9/10, ACKR1, CX3CR1, CXCR1/2/3/4/5/6, ADRA2A, ACKR3
			CXCL2 _ CCR3/6/8, ACKR1, CXCR1, CXCR2, GRM7
			CXCL3 _ CCR3/6/8, CXCR1, CXCR2, GRM7
			CXCL8 _ CCR1/2/3/4/5/6/7/8/9/10, SDC1/3, CX3CR1, ITGAM, CXCR1/2/3/4/5/6, XCR1, ACKR1, CDH5/79A, LPAR2, GRM7, ADRA2A,
			EDN1 _ EDNRA/B, ADGRL4, AR, MCAM, NPR3, CYSLTR1/2, AKT1, ADCY9, KEL
			EDN2_EDNRA/B, KEL
			EFNA1 _ RET, INSR, ROR1, NTRK1/2, PDGFRB, TYRO3, MERTK, EPHA10, MST1R, ALK, RYK, AATK, AXL, DDR2, PTK7, LMTK2, CSF1R, FLT3, PDGFRA, NTRK3, LTK, TIE1, MUSK, ROR2, MET
			IFNL1 _ IFNLR1
			PLAU _ ITGA3/A5/AV/AM/B1/B5, VLDLR, MRC2, LRP1, IGF2R, ST14
			TNF _ SLC5A11, TRPV1, PTPRU
			TNFSF10 _ TNFRSF10B/C/D, RIPK1
			TNFSF13B _ TNFRSF17/13B/13C
Ileum only	Downregulated	73	ANXA1 _ GRM7, GRIN2A
			ARPC5 _ ADRB2, LDLR
			B2M _ KIR2DL1/3DL1, AR
			CALM1 _ CRHR1, SCN4A, TRPC3, GRM3/4/7, SCTR, PTH2R, KCNQ3, AQP6, MIP, GRIN2A, PLPP6, OPRM1; CALM2_PLPP6, TRPC3/V1, SCN4A, GRM7, AQP6, GRIN2A, KCNQ3
			CALR _ ITGA2B/3/V, LRP1, SCARF1, PDIA3, AR, BDKRB2
			CDH1 _ CDH2,
			DSC2 _ DSG2
			FAM3C _ CLEC2D
			GRN _ SORT1
			HLA-A _ KIR2DL1/2DL4/3DL3/3DL1, KLRC3
			HLA-B _ KIR3DL3, KIR3DL1, KIR2DL1, KLRC3, KIR2DL4
			HLA-C _ KIR2DL4/2DL1/3DL1/3DL3, KLRC3
			HSP90B1 _ AR
			LYZ _ ITGAL
			MDK _ TSPAN1, SDC1/3, GPC2, ITGA4/6, PTPRB, ITGB1, LRP1, ALK
			PSAP _ AR, GPR37/37L1
			RTN4 _ RTN4RL1
			SPTAN1 _ PTPRA
Colon and ileum	Upregulated	66	TNF_CD83/H11, F2RL1/F3, TNFRSF21, SPHK1, NR3C1, AKT1, TRADD, RIPK1, MYLK, INSR, PPARG, PIGR, GPR160, PTPRS, CELSR2
			CXCL3 _ CXCR3/4/5/R1, CCR1/2/4/5/7/9/10, XCR1, ADRA2A
			CXCL10 _ CX3CR1, CCR1/2/4/5/7/9/10, CXCR3/4/5/6, ADRA2A, XCR1
			CXCL2_CXCR6 / CCR9 / CCR1 / CXCR5 / XCR1 CXCR3 / CXCR4 / CCR2 / CCR5 / CX3CR1 / ADRA2A / CCR4 / CCR7 / CCR10
			ICAM1 _ ITGAX/L/M, IL2RG/RA, CDH5, CAV1
Colon and ileum	Downregulated	104	ADAM9 _ ITGA3/6/V, ITGB1/5
			ANXA1 _ LMTK2, ADRA2A, FPR3, CCR10, DYSF
			B2M _ PDIA3, KLRC1/2, KIR2DL3, LILRB2, HFE, CD247, IL2RA/RB/RG, CD1A/B, CD3G/D
			CALM1 _ KCNQ1/5, PDE1B/C, MYLK, RYR1, INSR, VIPR1, GP6, PTPRA, GLP2R, KCNN4, ESR1/2
			CALM2 _ KCNQ1/5, ESR1/2, TRPC1, MYLK, RYR1, PDE1B/C, INSR, GP6
			CDH1 _ ITGAE/B7, LRP5/6, MET, KLRG1
			HLA-A _ IL2RA, KIR2DL3/3DL2, KLRC1/2/4, LILRB2, IL2RB/RG, CD3G/D, APLP2
			HLA-B _ KLRC1/2/4, CD3G/D, LILRB2, CANX, KIR2DL3/3DL2
			HLA-C _ CD3G, KIR2DL3/3DL2, CD3D, KLRC1/2/4, LILRB2
			HSP90B1 _ ASGR1, TLR1/2/4/7, LRP1
			PI3 _ PLD2
			PSAP _ CELSR1, LRP1, CD1B, SORT1
			RTN4 _ NGFR, TNFRSF19, GJB2
			S100A6 _ ESR1
			SLPI _ CD4, PLSCR1/4
			TIMP2 _ ITGA3, ITGB1

Table listing the number of ligand–receptor interactions in infected immature enterocytes–immune cells intercellular network that are unique or shared between the ileal and colonic networks. The direction column indicates the direction of the expression change epithelial ligand driving each intercellular interaction. Interactions are indicated as follows: ligand_receptor1, receptor2, receptor3. Receptors belonging to the same class (e.g. calmodulins) are indicated as follows: CALM1/2/3.

**Fig. 5:**
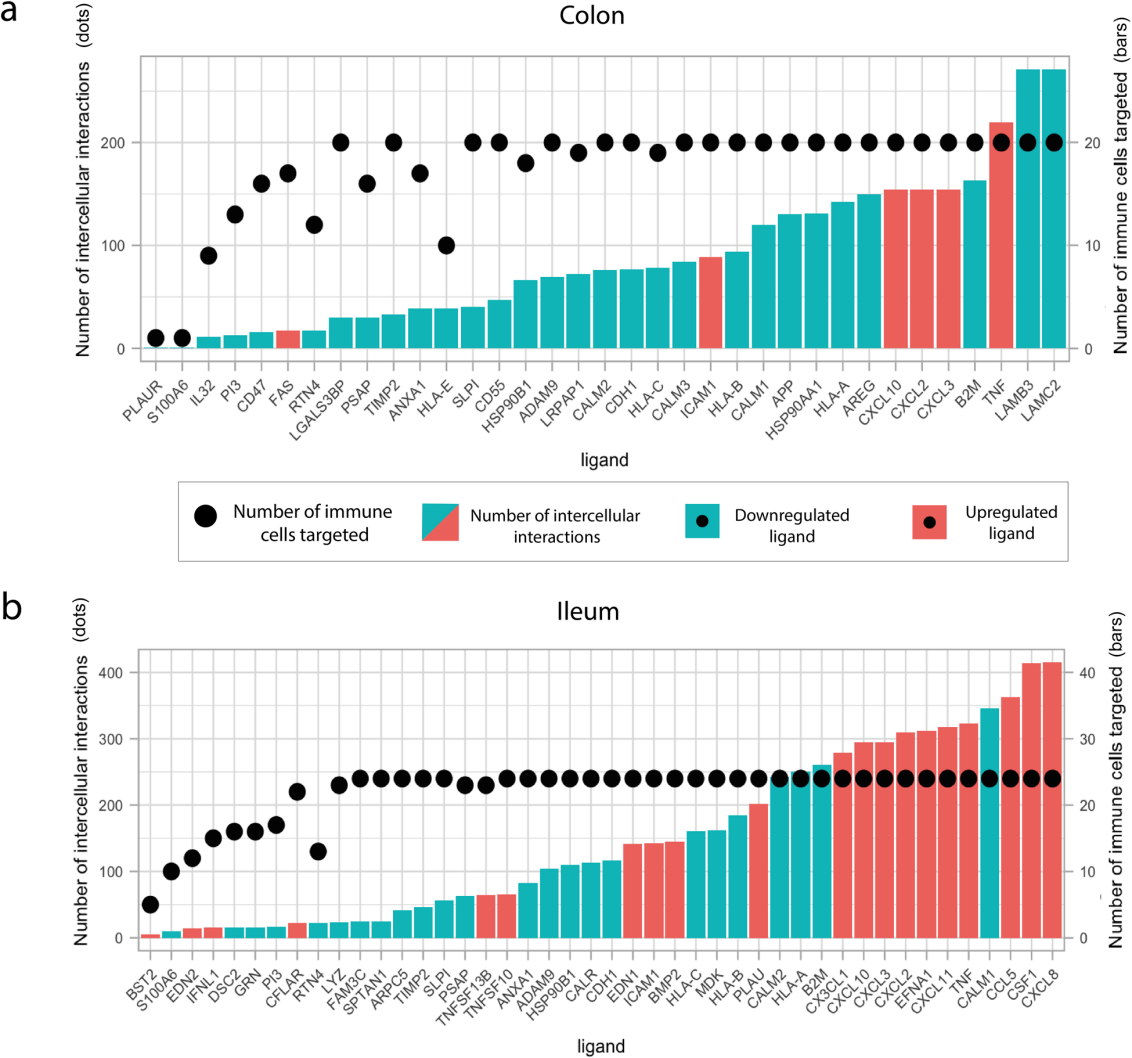
Overview of upregulated and downregulated ligands and ligand–receptor interactions between infected immature enterocytes and resident immune cells upon infection in the colon and ileum. **a**, **b** Bar plot showing the upregulated and downregulated ligands in the colonic (**a**) and ileal (**b**) infected immature enterocytes–immune cell network scored by number of interactions (height of the bar plot) and number of immune cells targeted (black dots). Upregulated ligands are shown in red and downregulated ligands in blue.

**Fig. 6:**
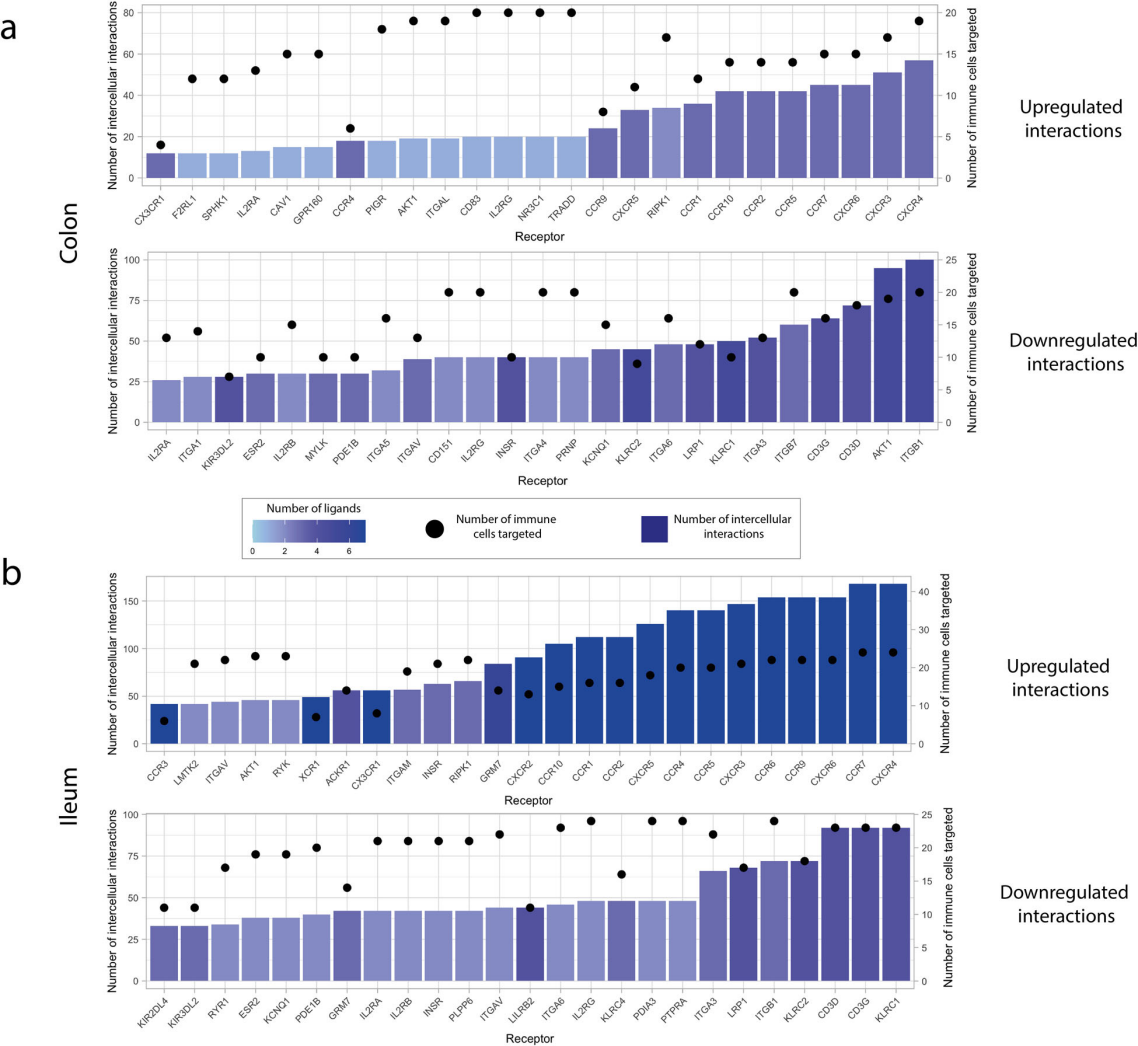
Receptors involved in intercellular interactions between colonic and ileal infected immature enterocytes and resident immune cells. **a**, **b** Bar plot showing the immune receptors targeted by upregulated (top graph) and downregulated (bottom graph) ligands in colonic (**a**) and ileal (**b**) infected immature enterocytes, scored by number of interactions (height of the bar plot) and number of immune cells targeted (black dots). The colour of the bar plots indicates the number of ligands targeting each of the receptors indicated. This plot only shows the top 25 receptors by number of interactions, and the full plot is available as Supplementary Fig. 6.

To understand which epithelial ligands and immune cell receptors were driving most epithelial–immune cell interactions, we scored ligands and receptors based on the number of interactions they were involved in (Fig. 1 and Methods). In both tissues, chemokines (CXCLs) and tumour necrosis factor alpha (TNF-α) were among epithelial ligands (Fig. 5a, b), and chemokine receptors (CXCR 3,4,5,6 and CCR 1,2,5,7,9,10) among the receptors on immune cells driving the highest numbers of upregulated interactions (Fig. 6), overall pointing towards an increased immune cell recruitment upon infection^36^. The high number of upregulated interactions driven by chemokines could be attributable to the widespread presence of several different chemokine receptors on immune cells (Supplementary Fig. 8).

**Fig. 7:**
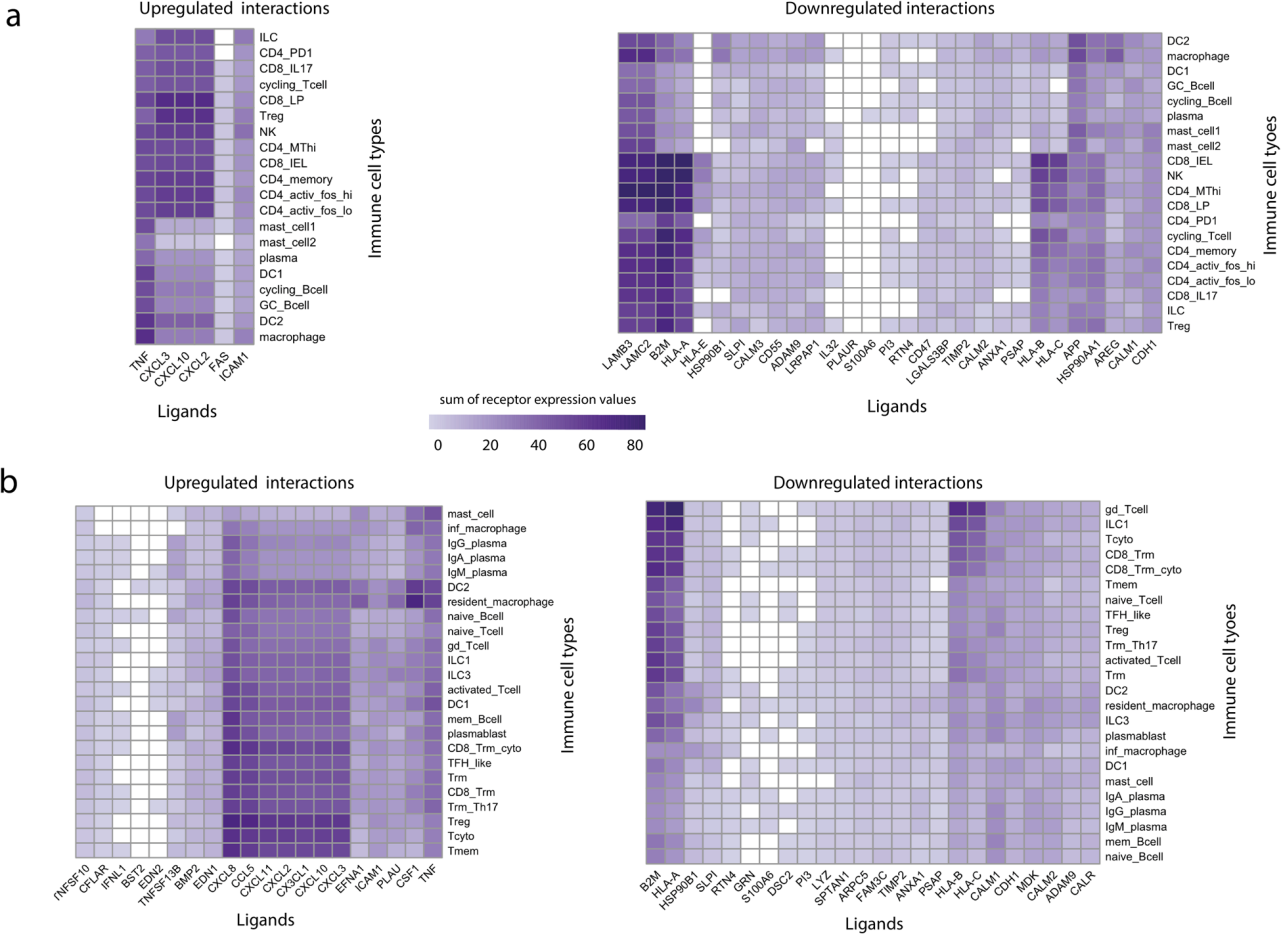
Ligands of infected immature enterocytes involved in the strongest up and downregulated interactions upon SARS-CoV-2 infection in the colon and ileum. **a**, **b** Heatmap showing the upregulated and downregulated interactions in the colon (**a**) and ileum (**b**) between intestinal epithelial ligands and resident immune cells upon infection of immature enterocytes with SARS-CoV-2. The strength of the interaction is expressed by accounting for the number of interactions between epithelial ligands and immune receptors and the level of receptor expression of immune cells. The strength of the interaction, named “sum of expression values”, is visualised using a colour gradient from white (weakeast interactions) to purple (strongest interactions). Abbreviations: *Ileum*: inf_macrophage infected macrophage, mast mast cell, CD8_Trm_cyto Resident memory cytotoxic T cell, DC2 dendritic cell 2, Trm Tissue-resident memory T cell, gd_Tcell Gamma delta (γδ) T cells, ILC Innate lymphoid cell, mem_Bcell memory B cell, naive_Bcell naive B cell, TFH_like T follicular helper cells, Trm_Th17 Tissue-resident memory Th17 cells, Treg Regulatory T cell, Tcyto Cytotoxic T cell, Tmem Memory T cells. *Colon*: ILC Innate lymphoid cell, CD8_IL17 IL-17+ CD8+ T cells, DC dendritic cells, GC_Bcell Germinal center B cells, CD4_PD1 mast mast cell, Treg Regulatory T cell, NK Natural Killer cell, CD4_MThi high mitochondrial CD4+ T cell, CD4_memory CD4+ Memory T cell, CD4_activ_fos_high activated CD4+ T cells (high/low c-fos), CD8_LP CD8+ lymphocyte-predominant cells, CD8_IEL CD8+ intraepithelial lymphocytes.

**Fig. 8:**
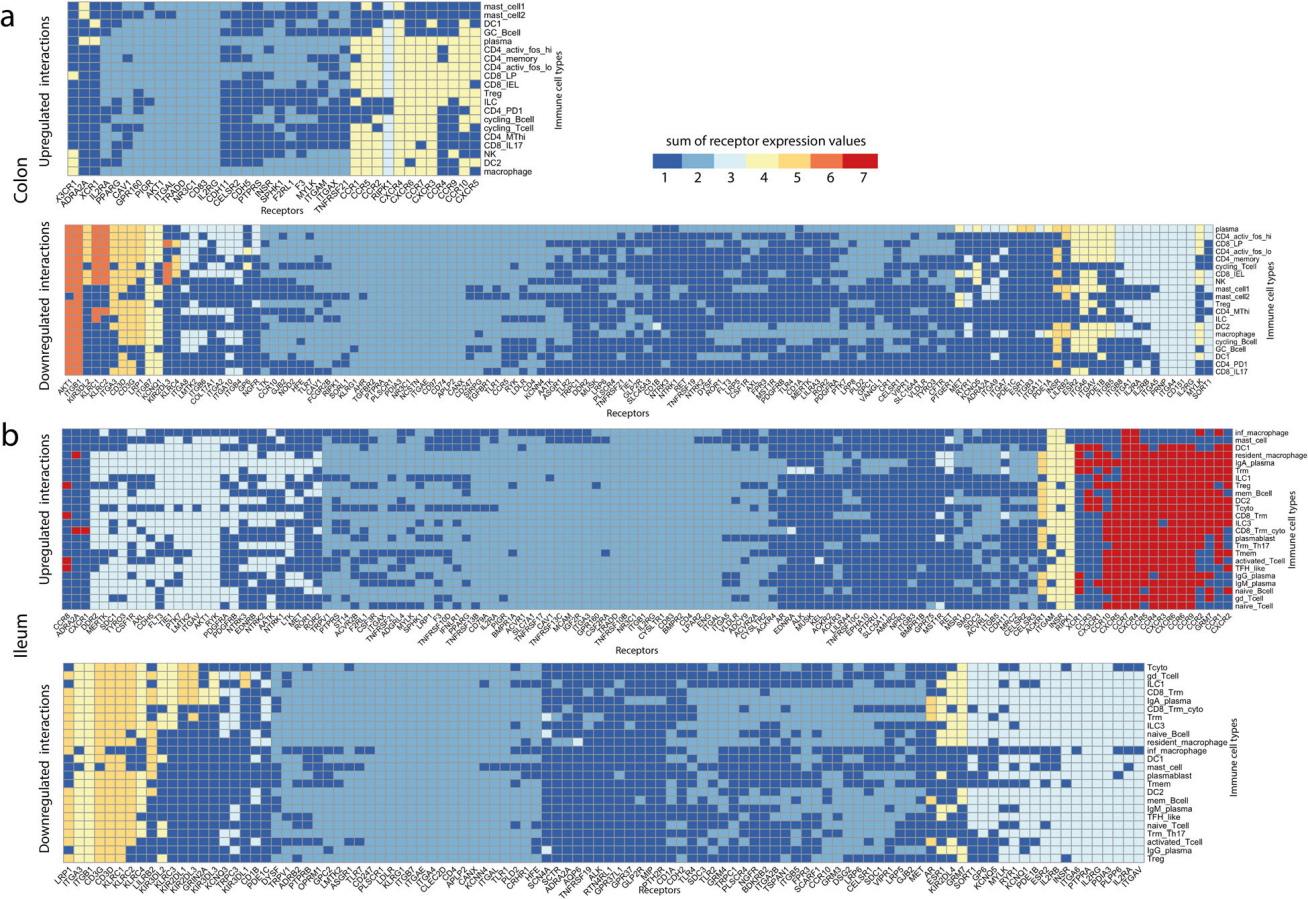
Receptors on immune cell types involved in the strongest up and downregulated interactions upon SARS-CoV-2 infection in the colon and ileum. **a**, **b** Heatmap showing the upregulated and downregulated interactions in the colon (**a**) and ileum (**b**) between receptors and resident immune cell types upon infection of immature enterocytes with SARS-CoV-2. The number of interactions in which each receiving receptor on immune cell types is involved is visualised using a colour gradient from blue (weakest interactions) to red (strongest interactions). Abbreviations: *Ileum*: inf_macrophage infected macrophage, mast mast cell, CD8_Trm_cyto Resident memory cytotoxic T cell, DC2 dendritic cell 2, Trm Tissue-resident memory T cell, gd_Tcell Gamma delta (γδ) T cells, ILC Innate lymphoid cell, mem_Bcell memory B cell, naive_Bcell naive B cell, TFH_like T follicular helper cells, Trm_Th17 Tissue-resident memory Th17 cells, Treg Regulatory T cell, Tcyto Cytotoxic T cell, Tmem Memory T cells. *Colon*: ILC Innate lymphoid cell, CD8_IL17 IL-17+ CD8+ T cells, DC dendritic cells, GC_Bcell Germinal center B cells, CD4_PD1 mast mast cell, Treg Regulatory T cell, NK Natural Killer cell, CD4_MThi high mitochondrial CD4+ T cell, CD4_memory CD4+ Memory T cell, CD4_activ_fos_high activated CD4+ T cells (high/low c-fos), CD8_LP CD8+ lymphocyte-predominant cells, CD8_IEL CD8+ intraepithelial lymphocytes.

To decipher which specific epithelial ligands and immune receptors were driving the strongest epithelial–immune cell interactions, ligands and receptors participating in epithelial–immune interactions can be scored based on the “sum of receptor expression” value, which takes into account the number of interacting receptors and the level of receptor expression in each immune cell type (Fig. 1 and Methods). In the colon, the strongest upregulated interactions involved the epithelial TNF-α binding to B cells, T cells (CD4/CD8+), NK cells, macrophages and DCs, as well as epithelial chemokines (CXCL2,3, 10) binding to T cells (CD4/CD8+) and NK cells (Fig. 7a). Similarly, in the ileum the strongest upregulated interactions involved epithelial chemokines binding to T cells (Treg, Tcyto, Tmem, CD8 Trm cyto) as well as TNF-α and colony stimulating factor 1 (CSF1) binding to macrophages and DCs (Fig. 7b). Receptors driving the strongest upregulated interactions were mainly chemokine receptors (CXCRs, CCRs) in both colon and ileum, and Receptor Interacting Serine/Threonine Kinase 1 (RIPK1) in the colon only (Fig. 8).

Finally, functional overrepresentation analysis of the participating upregulated epithelial ligands and receiving receptors on immune cells can help to understand the role of each of these epithelial–immune interactions driven by infection (Fig. 1). In line with the extensive overlap in upregulated intercellular interactions (Fig. 4a and Supplementary Fig. 8), most functions were shared between colon and ileum, and included chemotaxis (GPCR signalling, chemokine signalling), immunity (interleukin signalling), apoptosis (caspase activation) and angiogenesis (VEGFA-VEGFR2 pathway) (Supplementary Fig. 9). One colonic-specific function was related to pro-inflammatory responses (TNF signalling) (Supplementary Fig. 9a) and one ileal-specific function was related to stem cell renewal (BMP signalling) (Supplementary Fig. 9b).

Downregulated ligands in infected immature enterocytes upon infection as well as targeted receptors on immune cells were tissue-specific to a large extent, resulting in a large proportion of downregulated interactions being tissue-specific (73 unique to ileum, 125 to colon) (Figs. 2b, 4a, b). A detailed description of differences and similarities in ligands, receptors and downregulated intercellular interactions between colon and ileum can be found as Supplementary Results.

In both tissues, a high number of downregulated interactions was driven by the epithelial ligands human leukocyte antigens (HLA-A/B/C), beta-2-microglobulin (B2M) and calmodulin (CALM1/2) (Fig. 5a, b), and integrins (ITGs), KLRCs and LDL Receptor Related Protein 1 (LRP1) in both colon and ileum (Figs. 4a and 6). Additionally, uniquely in the colon, the highest number of downregulated interactions was driven by two epithelial-derived laminins (LAMC2, LAMB3) (Fig. 2c), and by AKT1 (Protein kinase B, PKB) present on immune cell types (Figs. 4a and 6).

When investigating the strength of these intercellular interactions, we found that HLA-s (HLA-A, B, C) and B2M targeting T cells (colon: CD4/CD8+, Tregs; ileum: Trm, Tregs, cytotoxic T cells), NK cells (colon only), ILCs and macrophages (ileum only) represented the strongest downregulated interactions in both colon and ileum (Fig. 7). Additionally, uniquely in the colon, laminins (LAMB3, LAMC2) targeting T cells and macrophages represented the strongest downregulated interactions (Fig. 7). Receptors driving the strongest downregulated interactions were AKT1 uniquely in the colon (Fig. 8a) as well as integrins, KLRCs and LRP1 in both colon and ileum (Fig. 8a, b)

Functional overrepresentation analysis of the participating downregulated epithelial ligands and receiving receptors on immune cells (Fig. 1 and Methods) revealed shared functions related to antigen processing and cross-presentation (MHC class I–mediated), phagocytosis (endoplasmic reticulum (ER) phagosome pathway, signalling by RHO GTPases) and cell–cell communication (immunoregulatory interactions between a lymphoid and non-lymphoid cell) in both tissues, possibly suggesting decreased epithelial–immune cell crosstalk functions related to the activation of the innate and adaptive immune response^37^ (Supplementary Fig. 9). Furthermore, several colon-specific functions were related to the extracellular matrix (ECM) organisation and integrin cell surface interactions, which play an important role in processes critical to inflammation, infection, and angiogenesis, thereby suggesting a negative regulation of these vital interactions uniquely in the colon^38^ (Supplementary Fig. 9a). The only function uniquely overrepresented in the ileum was transcriptional regulation by MECP2 (Supplementary Fig. 9b), whose expression has been shown to play a role in intestinal morphology and function^39^.

**Fig. 9:**
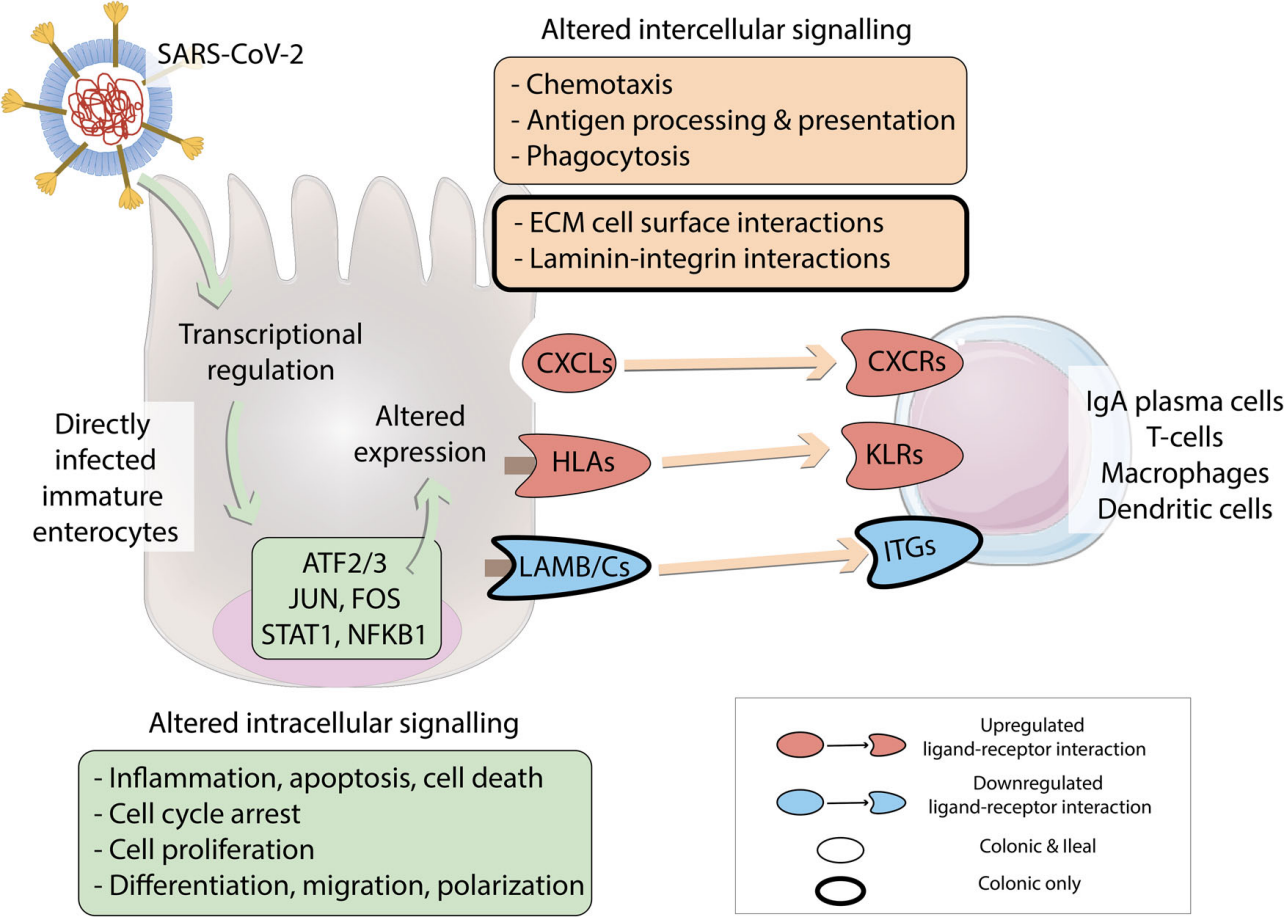
Overview of intracellular and intercellular signalling upon SARS-CoV-2 infection of colonic and ileal immature enterocytes and resident immune cells. SARS-CoV-2 directly infects colonic and ileal immature enterocytes. Upon infection, transcription factors ATF2/3, JUN, FOS, STAT1 and NFKB1 are modulated, resulting in altered intracellular signalling pathways and altered ligands expression, including upregulation of chemokines (CXCLs) and human leukocyte antigens (HLAs), and downregulation of laminins (LAMB/Cs). Altered ligands expression drives differential intercellular interactions between epithelial ligands and immune cell receptors (chemokine receptors (CXCRs), killer cell lectin-like receptors (KLRs) and integrins (ITGs)) expressed on immune cells.

In conclusion, using our framework, we pinpointed tissue-specific and shared epithelial ligands and immune cell receptors participating in the intercellular signalling through which SARS-CoV-2 infected epithelial cells can affect the inflammatory responses of various immune cell types during infection (Fig. 9).

### Implication of epithelial ligands in the inflammatory process

The analysis of our integrated framework of intracellular and intercellular signalling networks in intestinal epithelial cells upon infection has helped point out several differentially expressed ligands participating in epithelial–immune interactions potentially playing a role in driving the inflammatory process upon SARS-CoV-2 infection. To validate their importance during immune reactions, we integrated these predictions with independent data from three previously published studies (Fig. 1).

First, by comparing the differentially expressed ligands upon SARS-CoV-2 infection to DEGs in human colonic organoids exposed to inflammatory cytokines^40^, we identified 24 ligands whose expression change is regulated by cytokines during intestinal inflammation (Table 4). These ligands are likely to contribute to the inflammatory responses upon infection. Next, by comparing ileal and colonic ligands to data from ImmunoGlobe, a manually curated intercellular immune interaction network^41^ and ImmunoeXpresso, a collection of cell–cytokine interactions generated through text mining^42^, we identified 12 ligands previously known to influence immune cell populations (Table 4). The full list of affected immune cell types for each epithelial ligand is available in Table 4. Finally, to understand which ileal and colonic ligands could explain blood cytokine level changes of COVID-19 patients via direct immune cell regulation, we used data from^5^, and identified six ligands capable to create the detected blood cytokine levels during infection (Table 4).

**Table 4:** Key differentially expressed ligands produced by infected immature enterocytes drive the inflammatory process upon SARS-CoV-2 infection.

Ranking	Ligand	Ligand description	Organoid type	Expression change upon SARS-CoV-2 infection	Regulation by cytokines	Known to affect immune cells	Directly explain patients’ blood cytokine levels
1	CXCL2	C-X-C Motif Chemokine Ligand 2	Colonic, Ileal	Up	IFNG, TNF, IL-17, IL-22 (up)	Neutrophils (1,2), fibroblasts, T cells, NK cell and CD8a+ DCs (1), leukocytes (2)	✓
1	CXCL3	C-X-C Motif Chemokine Ligand 3	Colonic, Ileal	Up	TNF, IL-22 (up)	Neutrophils, fibroblasts (1), T cells (2)	✓
1	CXCL10	C-X-C Motif Chemokine Ligand 10	Colonic, Ileal	Up	IFNG, TNF (up)	DC, Th1, NK cells, B cells, monocytes (1), and 29 additional immune cell types (2)	✓
1	CSF1	Colony stimulating factor 1	Colonic	Up		35 immune cell types (2)	✓
1	CXCL11	C-X-C Motif Chemokine Ligand 11	Ileal	Up	n.d. (3)	DC (1,2), B cells, NK cells, Th1, monocytes, macrophages (1), lymphocytes, T cells, CD8+ alpha/beta T cell (2)	✓
2	TNFSF13B	TNF Superfamily Member 13b	Ileal	Up	n.d.	B cell, T cell, follicular B cell, naïve B cell, Th17, neutrophils, monocytes (2)	✓
2	LAMC2	Laminin Subunit Gamma 2	Colonic	Down	TNF, IL-22 (up)		
2	CCL5	C-C Motif Chemokine Ligand 5	Ileal	Up	n.d. (3)	T cells, basophils, eosinophils, macrophages, monocytes, NK cells, DC, Memory T cells, Th1 and Th2 (1), and 23 additional immune cell types (2)	
2	CX3CL1	C-X3-C Motif Chemokine Ligand 1	Ileal	Up	n.d. (3)	Monocytes, T cells, neutrophil, NK, DC, Mast cells and microglia (1), and 19 additional immune cell types (2)	
2	CXCL8	C-X-C Motif Chemokine Ligand 8	Ileal	Up	n.d. (3)	Neutrophils, macrophages, basophils, naïve T cells, CD8+ T cells, monocytes (1), and 44 additional immune cell types (2)	
3	ICAM1	Intercellular Adhesion Molecule 1	Colonic, Ileal	Up	IFNG, TNF, IL-22 (up)		
3	IL32	Interleukin 32	Colonic	Down	IFNG, TNF (up)		
3	AREG	Amphiregulin	Colonic	Down	IFNG (up), IL-13 (down)		
3	TNF	Tumour Necrosis Factor	Colonic, Ileal	Up	TNF, IL-22 (+)	Non-specific: 129 immune cell types (2)	
3	B2M	Beta-2-immunoglobulin	Colonic, Ileal	Down	IFNG (up)		
3	HLA-A	Major Histocompatibility Complex, Class I, A	Colonic, Ileal	Down	IFNG (up)		
3	HLA-B	Major histocompatibility complex, class I, B	Colonic, Ileal	Down	IFNG (up)		
3	LAMB3	Laminin Subunit Beta 3	Colonic	Down			

Table showing a list of top-ranked differentially expressed ligands in infected immature enterocytes which were identified to drive inflammation upon SARS-CoV-2 infection. The ranking of the ligands was performed using multiple criteria as explained in the Methods. ‘Organoid type’ indicates whether the expression change of the ligand was found in ileal or colonic infected immature enterocytes upon SARS-CoV-2 infection, respectively. ‘Expression change upon SARS-CoV-2 infection’ indicates the direction of expression change of the ligand in infected immature enterocytes upon SARS-CoV-2 infection. ‘Regulation by cytokines’ indicates whether ligand expression was found to be regulated by cytokines during inflammation based on results from^40^. Ileal data was not available (n.d.) in this study, so no conclusion could be drawn for ileal ligands. ‘Known to affect immune cells’ indicates whether the ligand was found to be regulated by immune cells using data from ImmunoGlobe^41^ and ImmuneXpresso^42^ databases. ‘Directly explain patient blood cytokine levels’ indicates whether the ligand was found to directly regulate blood cytokine levels in COVID-19 patients from^5^.

Using this assessment, we were able to rank the differentially expressed ligands for their importance in the inflammatory process, and subsequently listed the 18 highest ranked ligands, for which there is strong evidence of their role in epithelial–immune cell interactions during the inflammatory SARS-CoV-2 disease response (Table 4). These ligands included CSF1, various chemokines (CXCL10, CXCL11, CXCL2, CXCL3, CCL5, CX3CL1, CXCL8), TNFa and TNFSF13b, and ICAM1 among the upregulated ones; and various laminins (LAMC2, LAMB3), AREG, B2M), human leukocyte antigens (HLAs) (HLA-A, HLA-B) and IL32 among the downregulated ones.

### The intracellular and intercellular signalling are also altered in the upper airways in patients with moderate COVID-19

The presented integrated framework can also be applied to infected epithelial cell data from other organs to reveal the effects of viral infection on that specific organ’s function. To show the ease of applicability of our framework to other infected tissues, we have employed it to analyse the effect of SARS-CoV-2 on the intracellular and intercellular signalling of upper airway ciliated epithelial cells during moderate COVID-19 cases (Methods and Fig. 1). Functional analysis of the PPI layer of the intracellular signalling network of infected ciliated cells upon moderate COVID-19 revealed an alteration of pathways related to cell motility and migration, cell adhesion mediated by the ECM (laminin, non-integrins interactions), pro-inflammatory signalling pathways (Interleukin, MAPK, PI3K, NF-kB signalling), cell cycle arrest and intestinal homeostasis (Supplementary Fig. 12).

Intercellular interactions between upper airways ciliated epithelial cells and resident immune cells were mainly driven by upregulated ligands, particularly chemokines (CXCL1/3/6) and HLAs (HLA-A/B/C) binding to chemokine receptors (CCRs, CX(3)CRs) and CD3D/G, KLRs and KIRs (expressed on T-cells and NK cells), respectively (Supplementary Fig. 12 and Supplementary Fig. 13a, b). Interestingly, the strongest upregulated interactions were driven by chemokines and HLAs targeting T-cells (cytotoxic and regulatory), macrophages (resident and non-resident) and B cells, with a second cluster of slightly weaker interactions including NK, dendritic and mast cells (Supplementary Fig. 13c). Together, these results point towards increased recruitment and antigen presentation to these immune cell types. Functional overrepresentation analysis showed that upregulated interactions were related to chemokines/cytokines signalling, antigen processing and presentation, activation of the innate and adaptive immune system and general defense response (Supplementary Fig. 12).

Conversely, downregulated interactions were driven by epidermal growth factor receptor (EGFR) binding to several different receptors, and the ECM protein Tenascin C (TNC) binding to integrins (ITGs) on immune cells (Supplementary Fig. 12 and Supplementary Fig. 13a, b). Downregulated interactions involved most immune cells, with the strongest interactions targeting non-resident macrophages and regulatory T cells (Supplementary Fig. 13c). Functional overrepresentation analysis showed that downregulated interactions were involved in haemostasis and cell adhesion processes mediated by the ECM components (integrin, laminin, syndecan) (Supplementary Fig. 12).

## Discussion

In this work, we have developed an integrated framework to model how altered intracellular signalling in epithelial cells drives a different epithelial–immune interactome upon infection. As a proof of concept study, we first applied this model to highlight the putative role of the gut during the immune response following SARS-CoV-2 infection, showing how several intracellular and intercellular mechanisms are affected, with key differences between colon and ileum. A visual schematic of our key findings can be found in Fig. 9. Additionally, we proved the applicability of this framework to other tissues of interest by analysing intra and intercellular interactions of the upper airway epithelium in moderate COVID-19 patients, confirming many of the findings highlighted in the literature, and pointing out key cell–cell interactions of interest.

SARS-CoV-2 has been shown to actively infect and reproduce in the human gut and in human gastrointestinal organoids^10,11,13^. However, the exact effect of intestinal inflammation and the role of epithelial–immune interactions in the hyperinflammatory immune response (“cytokine storm”) characterising many COVID-19 patients are not known^5,6^. Accurately modelling these interactions could help identify potential targets that are key to selectively disrupt such cell–cell interactions underlying extreme inflammatory conditions during SARS-CoV-2 infection. This would be extremely important given the failure of most randomised control trials associated with pro-inflammatory drug candidates for COVID-19^43^.

The altered epithelial–immune cell crosstalk during SARS-CoV-2 infection has been explored within the nasopharynx and lungs using scRNA seq data^44^. This study found stronger epithelial–immune cell interactions in critically ill patients based on ligand–receptor expression profiles, highlighting the importance of the crosstalk between infected cells and local immune cells in the disease course. However, to our knowledge, no prior study has been carried out so far to investigate the effect of viral infection in host intestinal cells, and the role and contribution of intestinal epithelial cell–immune cell crosstalk during SARS-CoV-2 infection.

In this work, we developed an integrated pipeline to model the effect of intracellular signalling perturbation in epithelial cells on the epithelial–immune interactome in the gut. As a proof-of-concept, we exploited our previously published data on SARS-CoV-2 (BavPat1/2020) infection in intestinal organoids^13^ to investigate the effect of SARS-CoV-2 proteins and potential miRNAs on ileal and colonic epithelial cell intracellular signalling and function. We added in a distinguishable way the analysis of these potential miRNAs encoded by SARS-CoV-2, as previous studies highlighted the regulatory role of similar miRNAs produced by RNA viruses and their ability to downregulate host genes and affecting host functions^45–47^. Furthermore, we modelled how specific epithelial ligands, whose expression was altered upon infection, were driving specific epithelial–immune interactions *via* their altered binding to receptors expressed on resident immune cell populations^20,21^.

While our previous data pointed towards immature enterocytes as the prime target of SARS-CoV-2 infection, the application of our integrated pipeline allowed us to model how this epithelial population, when directly infected, also drives the majority of interactions with gut resident immune cells stemming from their differentially regulated ligands by SARS-CoV-2 (Fig. 2a). Upon infection of immature enterocytes, intracellular signalling pathways were altered, with a direct effect on pathways of inflammation, apoptosis, cell survival and cell death (Fig. 3). Pathways related to cell cycle (negative regulation of G2/M transition) and cell proliferation were also altered upon infection (Fig. 3), in line with a previous phosphoproteomics study finding a correlation with cell cycle arrest upon SARS-CoV-2 infection^48^. Finally, pathways involved in cell differentiation, cell migration and epithelial polarisation were also modulated upon infection (Fig. 3), which to our knowledge no other study had highlighted before.

By using available ligand–receptor interaction data, we aimed to understand how infected gut epithelial cells recruit resident immune cell populations to find key interactions driving the immune response during infection. Our analysis revealed that IgA plasma cells were the immune cell population with the highest number of cell–cell interactions upon infection, with the highest number of epithelial–immune interactions driven by downregulated epithelial ligands (29) in the colon, and upregulated epithelial ligands (20) in the ileum (Fig. 2a). A possible explanation for these observed tissue-specific differences and on the role of IgA plasma cells can be found in the Supplementary Discussion section.

By further analysing the specific ligand–receptor interactions driving epithelial–immune crosstalk upon SARS-CoV-2 infection, we could observe that strong upregulated interactions upon infection were mostly shared by both colon and ileum, and were represented by chemokine and TNF-α driven interactions, possibly reflecting a general effect of the inflammation process (Figs. 6 and 7). Functional analysis highlighted a relation to proinflammatory signalling pathways, including TNF-α signalling, interleukin signalling and chemotaxis via GPCR signalling, overall suggesting an increasing recruitment and cell adhesion of these immune cell populations upon infection (Supplementary Fig. 9). Notably, four chemokine receptors identified by our study (CXCR6 in the ileum, CCR1/2 and CCR9 in both ileum and colon) are coded in a genomic region found to be a COVID-19 risk locus on chromosome 3, further validating our predictions^49^.

Conversely, we could observe that strong downregulated interactions were driven by epithelial HLAs (HLA-A, B, C) and B2M, a subcomponent of the major histocompatibility complex I (MHC I) in both tissues (Fig. 6). According to our analysis, these ligands were mainly binding to KLR receptors, which are mainly presented on NK cells (Fig. 6, Supplementary Fig. 8). Downregulation of HLA-KLR interactions may represent an immune evasion mechanism^50^ that a recent study proposed as a way SARS-CoV-2 protein ORF8 uses to escape host immune surveillance^51^.

Uniquely in the colon, strong downregulated interactions were driven by epithelial laminins (LAMB3 and LAMC2) and integrins, with T cells and macrophages as the main immune cell types targeted upon infection (Figs. 6 and 7). Laminin–integrin binding contributes to focal adhesion of immune cells to the inflamed tissue^52^, and downregulation of laminins could represent an additional strategy for immune evasion following viral infection uniquely in the colon. Furthermore, laminins are known to play a role in shaping the architecture of intestinal mucosa, and an altered expression has been observed in Crohn’s disease, a type of IBD, driven by pro-inflammatory cytokines TNF-α and IFN-γ^53–55^. Finally, an additional mechanism that SARS-CoV-2 may use to evade the immune response via the downregulation of calmodulin-phosphodiesterases interactions is further discussed in the Supplementary Discussion section.

With our integrated framework, we provided a key tool to study the effect of intracellular signalling perturbation in gut epithelial cells driving differential epithelial–immune interactions. By applying this workflow on SARS-CoV-2 infected organoids scRNA seq data, we confirmed many of the previous findings about SARS-CoV-2 infection, including the induced pro-inflammatory responses driven by chemokines and the role played by T cells (Fig. 9). Additionally, we uncovered mechanisms by which SARS-CoV-2 may evade the immune responses by interfering with epithelial–immune cell connections. Such mechanisms include downregulation of antigen presentation mediated by HLAs-KLR interactions and of focal adhesion pathways mediated by laminin–integrins interactions (Fig. 9).

In this work, we highlighted a set of intestinal epithelial ligands and immune cell populations implicated in altered epithelial–immune interactions during SARS-CoV-2 infection, which could potentially drive the excessive inflammatory processes seen in severe COVID-19 patients (Table 4). Further experimental validation of bioinformatics predictions is key to validate these processes and the main molecules and cell types involved. To this end, intestinal organoids represent an excellent in vitro model to enable such validations^56^. Currently, introduction of immune cells to an organoid system is a challenging task. Yet, a recent study where human intestinal CD4+ T cells have been co-cultured with human intestinal organoids^57^, may represent a promising set-up for future studies to investigate epithelial–immune cell interactions during SARS-CoV-2 induced inflammation in the gut. As reviewed recently by^58,59^, such co-culture systems could be excellent to study intestinal host-microbe interactions, including the detailed experimental analysis of SARS-CoV-2 infection.

Nonetheless, our integrated workflow presents some limitations. When constructing the intracellular causal network, the effect of SARS-CoV-2 proteins towards human binding partners was always considered as inhibitory. However, this is not always the case. In the future, with increasingly available data, a more refined model could be generated. Furthermore, two different single cell transcriptomics datasets were used for colonic and ileal immune cell populations, due to the unavailability of both datasets from the same experiment. Similarly, IBD uninflamed data and healthy data were used for the ileum and colon respectively, as healthy control scRNAseq immune cell data for both tissues was not available at the time of the analysis. Finally, the a priori resources used to infer the intracellular and intercellular interaction networks may have some intrinsic limitations associated with them^60^. Specific tools such as LIANA (LIgand–receptor ANalysis frAmework; https://github.com/saezlab/liana) could be used in the future to compare across several resources available, helping to choose the one(s) providing the best overall prediction.

With our integrated workflow, we established a computational method to evaluate the effect of viral infection on host intestinal epithelial cell functions and how this consequently modulates the epithelial–immune crosstalk and immune activation. To demonstrate its applicability to other tissues, we analysed the intracellular and intercellular signalling of upper airway epithelial data of moderate COVID-19 cases. Although no specific information about the infecting SARS-CoV-2 strain was available, we were able to confirm several general findings related to COVID-19 infection previously highlighted in the literature. Following infection of ciliated cells, pro-inflammatory signalling pathways (interleukin, MAPK and NF-kB signalling) were altered, indicating an activated immune status, as previously reported^48,61–63^. Upregulated interactions were driven by chemokines binding to T cells (cytotoxic and regulatory) and macrophages, in line with the known role of chemokines in driving inflammation and immune cell recruitment^14,64^ and the role played by T cells macrophages in the innate and adaptive immune response to SARS-CoV-2^65,66^. Additionally, upregulated interactions were also driven by HLAs, which are part of the MHC I complex, whose association with symptom severity has been previously highlighted^67^. Interestingly, the upregulation of intercellular interactions driven by HLAs is the opposite of the effect found in the intestine, which may represent a key difference in the response to SARS-CoV-2 between these two tissues. Finally, we found strong downregulated interactions between Tenascin C (TNC) and integrins, which were related to cell adhesion processes mediated by the ECM components. This is in line with a study finding that proteins associated with focal adhesion and the ECM receptors were decreased in COVID-19 lung tissue, which could indicate a dysregulation of the extracellular microenvironment in this tissue, revealing a possible mechanism of SARS-CoV-2-related lung damage^68^.

To conclude, we demonstrated that this workflow is not limited to the gut, but it can be easily applied to other organs and cell types (e.g. lung, kidney, heart), provided the right input data is available. The workflow is translatable and both the epithelial and the immune component are replaceable. Furthermore, the presented workflow is transferable to understand any past or future infectious disease, when transcriptomic data of infected and control tissue and viral interactors are available. In this way, our workflow could potentially and efficiently be used in any other infection studies to shed light on the potential intervention points between immune cells and infected cells.

## Methods

### Intercellular analysis

#### Input data

##### Intestinal epithelial cells

Single cell transcriptomics data of colonoids and enteroids infected with SARS-CoV-2 (BavPat1/2020 strain) was obtained from^69^. Single cell transcriptomics data of upper airway epithelial cells from moderate COVID-19 patients were obtained from^44^. Strain-level information about the infecting SARS-CoV-2 variant was not available in this study. The R packages ‘Mast’ and ‘Seurat’ were used to identify differentially expressed genes upon infection with SARS-CoV-2 for each epithelial cell type^70,71^. Specifically, directly infected or bystander cells from intestinal organoids treated with SARS-CoV-2 for 24 h were compared with the equivalent cell type from uninfected organoids. For the airway analysis, ciliated airway epithelial cells from moderate COVID-19 patients were compared with the equivalent cell type in control patients. This cell type was chosen as it is the most prevalent and most affected by SARS-CoV-2 infection together with secretory cell types^44^. Any genes with adjusted *p* value ≤0.05 and |log2 fold change (FC)| ≥ 0.5 were considered significantly differentially expressed. Differential expression could only be calculated for cell types within a condition where data was available from ≥3 individual cells.

##### Intestinal resident immune cells

Single cell expression data from ileal and colonic resident immune cells was obtained from^20^ and^21^, respectively. For the analyses, data from healthy samples and uninflamed Crohn’s disease samples was used for colonic and ileal immune cell populations, respectively. Single cell expression data of upper airway immune cells from moderate COVID-19 patients was obtained from^44^.

Immune cell populations were identified through annotated clustering from^20,21,44^. Cell type labels were maintained as originally published. Following removal of all genes with count = 0, normalised log2 counts across all samples (separately for each cell type) were fitted to a gaussian kernel^72^. All genes with expression values above mean minus three standard deviations were considered as expressed genes for the given cell type in the given intestinal location. For the intercellular ligand–receptor predictions in the colon and ileum, a representative collection of immune cells relevant in gut inflammation and SARS-CoV-2 infection based on previous literature was selected, which included all macrophages, T cells, B cells, plasma cells, Innate Lymphoid Cells (ILCs), mast cells and a representative group of dendritic cells (DCs)^20,21,49,66,73^. For the ligand–receptor predictions of the upper respiratory tract, all immune cell types for which information was available were used in the analysis.

#### Defining ligand–receptor interactions between cell types

A list of ligand–receptor interactions was obtained from OmniPath on 23 September 2020 using the ‘OmnipathR’ R package^18^. Source databases used to retrieve the ligand–receptor interactions through OmnipathR included six independent resources (CellPhoneDB, HPMR, Ramilowski 2015, Guide2Pharma, Kirouac 2010, Gene Ontology)^25,74–78^. No weighing was performed on ligand–receptor interactions, and protein complexes were dealt with by including each of their individual proteins in the list.

Ligand–receptor interactions (intercellular interactions; full list available at https://github.com/korcsmarosgroup/gut-COVID) were predicted between epithelial cells types and resident immune cells according to the following conditions:

1. The ligand is differentially expressed in the epithelial cell (upon SARS-CoV-2 infection—in directly infected or bystander cells of the colon and ileum).
2. The receptor is expressed in the immune cell in the relevant dataset (ie, ileal or colonic immune cells).
3. The ligand–receptor interaction is present in OmniPath.

For the gut analysis, intercellular interactions were defined separately for directly infected epithelial cells and bystander epithelial cell populations in the ileum and in the colon. Enteroid epithelial data was paired with ileal immune cell data^20^, while colonoid epithelial data was paired with colonic immune cell data^21^. For the upper airway analysis, ciliated cell data of moderate COVID-19 samples was paired with the same cell type of control samples^44^. Intercellular interactions were defined between every possible pair of epithelial cells and immune cells for each condition. Interactions derived from upregulated ligands (“upregulated interactions”) were evaluated separately from interactions derived from downregulated ligands (“downregulated interactions”).

#### Scoring of ligands, receptors and immune cell types involved in ligand–receptor interactions

To assess the importance of specific ligands, receptors and immune cell types, additional parameters were computed using the ligand–receptor network. First, the number of interactions between each epithelial and immune cell type was computed by summing up all the possible interactions between each differentially expressed epithelial ligand and each of the receptors expressed by the specific immune cell type. Second, the number of immune cell types involved in each ligand–receptor pair was computed by counting the number of different immune cell types which were expressing the receiving receptor. Third, for each ligand, a “sum of receptor expression value” was computed for each interacting immune cell type, based on the number of interacting receptors and the mean expression level of the interacting receptors.

#### Data visualisation

Venn diagrams were generated using the ‘gplots’ R package. Heatmaps were generated using the ‘ggplot2’ and ‘pheatmap’ packages^79,80^. Barplots were generated with the ‘ggplot2’ package. Network visualisations were done using Cytoscape (version 3.8.2) (Shannon et al. 2003; Su et al. 2014). All scripts used to generate these plots are available on the Github repository of the project (https://github.com/korcsmarosgroup/gut-COVID).

### Intracellular analysis

Two previously established tools were employed to predict the effect of SARS-CoV-2 infection on epithelial cells: ViralLink and CARNIVAL^17,19^. Both tools, using related but distinct methods, infer causal molecular interaction networks. These networks link perturbed human proteins predicted to interact with SARS-CoV-2 viral proteins or miRNAs, to transcription factors known to regulate the observed differentially expressed ligands in infected epithelial cells.

#### Input data

To reconstruct the intracellular causal networks, three different a priori interactions datasets were used. Information on viral proteins and their interacting human binding partners was obtained from the SARS-CoV-2 collection of the IntAct database on 1st October 2020^81,82^. Predicted SARS-CoV-2 miRNAs and their putative human binding partners were obtained from^45^. Intermediary signalling protein interactions known to occur in humans were obtained from the core protein-protein interaction (PPI) layer of the OmniPath collection using the ‘OmnipathR’ R package on 7th October 2020^83^. Only directed and signed interactions were included. Interactions between human transcription factors (TFs) and their target genes (TG) were obtained from the DoRothEA collection using the DoRothEA R package on 7th October 2020^84^. Only signed interactions of the top three confidence levels (A, B, C) were included.

Normalised transcript counts and differentially expressed ligands were obtained from single cell transcriptomics data of (i) colonoids and enteroids infected with SARS-CoV-2 obtained from^13^, or (ii) upper airways samples of moderate COVID-19 patients from^44^, as previously described.

#### ViralLink pipeline

Intracellular causal networks were inferred using the ViralLink pipeline, as described in^17^. Briefly, a list of expressed genes in infected immature enterocytes (originally known as “immature enterocytes 2” (MMP7+, MUC1+, CXCL1+)) from SARS-CoV-2-infected ileal and colonic organoids^13^ or ciliated epithelial cells from moderate COVID-19 samples^44^ was generated from a normalised count table by fitting a gaussian kernel^72^. The list of expressed genes in the infected immature enterocytes population or COVID-19 ciliated epithelial cells was subsequently used to filter the a priori molecular interactions from OmniPath and DoRothEA, to obtain PPI and TF-TG sub-networks where both interacting molecules are expressed (described as “contextualised” networks). Transcription factors regulating the differentially expressed ligands were predicted using the contextualised DoRothEA TF—TG interactions and scored as described in^17^. Human binding proteins of viral proteins and viral miRNAs obtained from the IntAct database^81,82^ and^45^, respectively, were connected to the listed TFs through the contextualised PPIs using a network diffusion approach called Tied Diffusion Through Interacting Events (TieDIE)^85^. In this model, all viral protein—human binding protein interactions were assumed to be inhibitory in action, based on likely biological function, and given a lack of clear literature evidence of proven action. All viral miRNA—human binding protein interactions were set as inhibitory based on biological action of miRNAs^86^. The final reconstructed network contains “nodes”, which refers to the interacting partners, and “edges”, which refers to the interaction between nodes. Nodes include viral proteins and miRNAs, human binding proteins, intermediary signalling proteins, TFs and differentially expressed ligands. Edges include activatory or inhibitory interactions.

For ileal, colonic and upper airways data, separate networks were generated using the viral miRNA and viral protein as perturbations, and subsequently joined using the “Merge” function within Cytoscape to generate the final intracellular network. Nodes and edges were annotated according to their involvement in networks downstream of viral miRNAs or proteins. Further analyses were performed separately on the different layers of the network: miRNA specific, protein specific or shared nodes.

#### CARNIVAL pipeline

Intracellular causal networks were inferred using CARNIVAL and associated tools for analyses of expression data as described in^19^. For simplicity, we refer to the pipeline as described in^19^ as the CARNIVAL pipeline. Briefly, PROGENy is used to infer pathway activity from the log2 FC of the infected immature enterocytes 2 gene expression data^87^. Next, using the TF-TGs interactions (from DoRothEA) and the differential expression data from infected organoids, VIPER was used to score TF activity based on enriched regulon analysis^88^. Here, only the top 25 TFs regulating at least 15 target genes were taken forward, and a correction for pleiotropic regulation was included. Finally, CARNIVAL applied an integer linear programming approach to identify the most likely paths between human interaction partners of viral proteins or miRNAs and the selected TFs, through PPIs from OmniPath, considering the activity scores from PROGENy and VIPER. Viral protein—human binding protein interactions signs were specified to CARNIVAL as ‘inhibitory’, based on likely biological function, and given a lack of clear literature evidence of proven action. All viral miRNA—human binding protein interactions were also set as inhibitory based on biological action of miRNAs^86^.

### Network functional analysis

Functional overrepresentation analysis was performed on the networks constructed as above-mentioned using the R packages ‘ClusterProfiler’ and ‘ReactomePA’, for Gene Ontology (GO)^25^) and for Reactome^22–24^ annotations, respectively. For the intercellular network, the analysis was carried out separately for ligand–receptor intercellular interactions driven by either upregulated or downregulated ligands. A complete list of ligand–receptor interactions is available in the GitHub repository of the project (https://github.com/korcsmarosgroup/gut-COVID). For the upregulated interactions, a list of upregulated ligands and their connecting immune receptors was used as the test. For the downregulated interactions, a list of downregulated ligands and their connecting immune receptors was used. Where a list of ligands plus receptors contained <5 genes, it was excluded from the analysis. All ligands and receptors from the original ligand–receptor network used as prior knowledge input for the intercellular analysis was used as the statistical background.

For the intracellular network, the analysis has been done separately for each of the sub-networks (viral protein specific, viral miRNA specific, or shared). For each sub-network, a set of genes that were human binding proteins, intermediary proteins and TFs in the network (“PPI layer”) was used as a test list, and a set of all nodes from the original OmniPath PPI interaction network used as prior knowledge input for the intracellular analysis was used as the statistical background. For the Reactome pathway enrichment analysis the IDs were converted to Entrez Gene ID within the ‘ReactomePA’ package. Functional categories with adjusted *p* value ≤ 0.05 and with gene count >3 were considered significantly overrepresented.

### Selection of ligands involved in the inflammatory process

To show how our approach could help point out specific epithelial-derived ligands driving the inflammatory process upon SARS-CoV-2 infection, the list of differentially expressed ligands in infected immature enterocytes in both colon and ileum was validated using independent data from three previously published studies. To identify ligands whose expression was induced by cytokines, ligands were compared to DEGs in human colonic organoids exposed to cytokines from^40^. To identify ligands already known to influence immune cell population, ligands were compared to two databases: ImmunoGlobe, a manually curated intercellular immune interaction network^41^, and ImmunoeXpresso, a collection of cell–cytokine interactions generated through text mining^42^. Finally, to identify ligands that could directly explain blood cytokine level changes in COVID-19 patients via direct immune cell regulation, ligands were compared to the data from a large dataset we recently manually compiled using COVID-19 patient publications^5^.

## Supplementary information

Supplementary Text and Figures

## Data Availability

The necessary input data for the workflow and the full ligand–receptor interaction tables are available in the GitHub repository of the project (https://github.com/korcsmarosgroup/gut-COVID). All other relevant data is in the main text and in Supplementary files.

## Code availability

The code of the entire workflow is available in the GitHub repository of the project (https://github.com/korcsmarosgroup/gut-COVID).
