# Supplementary material for: Mapping the epithelial–immune cell interactome upon infection in the gut and the upper airways
Source: NPJ Syst Biol Appl. 2022 May 2;8:15. doi: 10.1038/s41540-022-00224-x (PMC9061772; doi:10.1038/s41540-022-00224-x)
Supplement: Supplementary file 1 — Supplementary Text and Figures [file 41540_2022_224_MOESM1_ESM.pdf]

Mapping the epithelial-immune cell interactome upon infection in the gut and the upper airways

Martina Poletti, Agatha Treveil, Luca Csabai, Leila Gul, Dezso Modos, Matthew Madgwick, Marton Olbei, Balazs Bohar, Alberto Valdeolivas, Denes Turei, Bram Verstockt, Sergio Triana, Theodore Alexandrov, Julio Saez-Rodriguez, Megan L. Stanifer, Steeve Boulant, Tamas Korcsmaros

Supplementary Results

Intracellular signalling networks upon SARS-CoV-2 infection

Colonic and ileal intracellular networks generated using ViralLink were similar in terms of size and network characteristics for ileum and colon, when considering the diameter, characteristic path length, average number of neighbours, and number of molecular entities (nodes, miRNAs, genes or proteins) and molecular interactions (edges, activatory or inhibitory). The colonic network was made of 1423 nodes and 9971 edges, and had a network diameter of 10, characteristic path length of 4 and average number of neighbours of 14 (**Supplementary Figure 2**). Additionally, the ileal network was made of 1316 nodes and 7935 edges, and had a network diameter of 9, characteristic path length of 4 and average number of neighbours of 12 (**Supplementary Figure 2**). In the colon, we found 47 viral proteins/miRNAs, 409 human binding proteins, 908 intermediary signalling proteins, 37 TFs and 22 differentially expressed ligands, while in the ileum we found 47 viral proteins or miRNAs, 394 human binding proteins, 810 intermediary signalling proteins, 37 TFs and 28 differentially expressed ligands (**Supplementary Figure 2**). Notably, upstream signalling was predicted for 22 out of the initial 35 differentially expressed ligands (29 down- and 6 up-regulated) in the colon, and for 28 out of 44 differentially expressed ligands (24 down- and 20 up-regulated) for the ileum (**Supplementary Figure 2**). These numbers are lower than those predicted to be differentially expressed upon infection by (Triana et al., 2021a), indicating that some ligands are not affected by direct upstream signalling

changes but by more complex mechanisms, or the original knowledge network used as input for the analysis did not contain information about such ligands (Menche et al., 2015) (**Supplementary Figure 1 and 2**).

Intracellular signalling networks generated using CARNIVAL were of much smaller sizes compared to those built with ViralLink (**Supplementary Figure 2**). This result is linked to the property of CARNIVAL to find the most optimal paths based on the given input constraints compared to ViralLink where all possible interactions are explored. Hence, CARNIVAL networks are very useful to understand specific molecular mechanisms and modulators upon SARS-CoV-2 infection.

The epithelial-immune interactome driven by SARS-CoV-2 regulated ligands in infected epithelial cells

Upregulated ligands of infected immature enterocytes upon infection were largely shared between colon and ileum **(Figure 2B)**. Shared upregulated ligands included mainly cytokines and chemokines (CXCL2/3/10 and tumor necrosis factor (TNF-a)) and the adhesion factor ICAM1. Interestingly, several additional chemokines (CSF1, CXCLs, TNFSFs) and adhesion factors (PLAU, EFNA) were upregulated in the ileum upon infection, which we did not find in the colon (**Figure 2B**). Additionally, 38 receptors on immune cells targeted by upregulated ligands in the colon were all shared with the ileum, and were mainly represented by chemokine receptors (CXCRs, CCRs) (**Figure 5**). Epithelial-immune interactions driven by upregulated ligands were also mostly shared in the colon and ileum (1 unique to colon, 219 unique to ileum, 66 shared) **(Figure 4C** and **Supplementary Figure 8)**.
Ileal-specific upregulated interactions were driven by Plasminogen Activator (PLAU), Ephrin A1 (EFNA1) and colony stimulating factor 1 (CSF1) binding to various receptors on immune cells, pointing towards an increased immune cell recruitment and adhesion **(Figure 4C** and **Supplementary Figure 8B).** Finally, we found one colon-specific upregulated interaction between epithelial Fas Cell Surface Death Receptor (FAS) binding to receptor-interacting serine/threonine-protein kinase 1

(RIPK), pointing towards increased cell death upon infection (**Figure 4C** and **Supplementary Figure 8A**).

Downregulated ligands in infected immature enterocytes were partially shared between colon and ileum, but were tissue-specific to a large extent (**Figure 2B**). Additionally, receptors on immune cells targeted by downregulated ligands were partially shared between colon and ileum (66), but several of them were tissue-specific (63 unique to colon, 38 unique to ileum) (**Figure 5**). In line with this, while some downregulated interactions in infected immature enterocytes were shared (104), a large proportion was tissue-specific (73 unique to ileum, 125 to colon) **(Figure 4C** and **Supplementary Figure 8).**

In both tissues, the highest number of downregulated interactions interactions was driven by epithelial ligands human leukocyte antigens (HLA-A/B/C))**,** beta-2-microglobulin (B2M) and calmodulin (CALM1/2) (**Figure 4A, 4B**). Interestingly, uniquely in the colon, the highest number of downregulated interactions was driven by two epithelial-derived laminins (LAMC2, LAMB3) (**Figure 4A**) and by AKT1 (Protein kinase B, PKB) (**Figure 6** and **Supplementary Figure 6**).

The epithelial-immune interactome driven by SARS-CoV-2 regulated ligands in bystander epithelial cells upon infection

In bystander cells, the number of ligands was much lower than in infected cells (**Supplementary Figure 1**). Nevertheless, similar effects could be found, with the highest number of upregulated interactions driven by epithelial chemokines (CXCL10/11) and TNF-a binding to chemokine receptors (CXCRs, CCRs) and TNF receptors (ileum only) on immune cells in both tissues (**Supplementary Figure 5 and 7)**. Additionally, strong upregulated interactions involved epithelial chemokines and various CD4+ and CD8+ T cells, B cells and ILCs in both colon and ileum **(Supplementary Figure**

11). Functional overrepresentation analysis revealed that these interactions were mainly related to recruitment of immune cells to the epithelium (GPCR signalling, chemokine signalling) and cell death/necrosis in both colon and ileum (**Supplementary Figure 10**).

In bystander cells, most downregulated interactions were driven by epithelial laminin (LAMA3) binding to integrins (ITGs) on immune cells in the colon only, while no downregulated interactions were found in the ileum (**Supplementary Figure 5**). In the colon, strongest downregulated interactions involved epithelial laminin LAMA3 and various CD4+ and CD8+ T cells, NK cells and macrophages **(Supplementary Figure 11A**). Functional overrepresentation analysis revealed that these interactions were mainly related to extracellular matrix organization (**Supplementary Figure 10A).**

Supplementary Discussion

IgA plasma cells were the immune cell population with the highest number of cell-cell interactions upon infection in both colon and ileum (**Figure 2A**). Notably, previous reports suggests that IgA is the main type of immunoglobulin induced by mucosal infection of SARS-CoV-2, stressing the importance of the crucial role played by IgA-mediated mucosal immunity in anti-SARS-CoV-2 infection (Sterlin et al., 2021).

Our analysis revealed that in the colon, most epithelial-immune interactions were driven by downregulated epithelial ligands (29), including laminins, HLAs and calmodulins, possibly suggesting a decreased antigen presentation and calcium-dependent activation of these cell types (**Figure 2A, 4A**). Conversely, in the ileum these interactions were driven by upregulated epithelial ligands (20), mainly cytokines/chemokines (TNF-a, CXCLs, CSF1) and adhesion factors (ICAM1, PLAU), possibly suggesting increased recruitment of these cell types to the epithelium (**Figure 2A, 4B**). Interestingly, the number of interactions with immune cells was not simply driven by the overall number of SARS-CoV-2 regulated ligands but by a few ligands presenting many different receptors on immune cells,

and their relative expression change following infection (**Figure 4A, 4B and Supplementary Figure 8**). Of note, the size of each immune cell population was not taken into account in this analysis (**Methods**). Extension of the study in this way could refine the importance of each type of ligand-receptor communication in mediating the overall downstream functional changes, leading to a better prediction of the effect size for each ligand-receptor combination. Nevertheless, the potential downregulation of cell-cell interactions with IgA plasma cells in the colon is an interesting avenue of further research.

Calmodulin genes (*CALM1, CALM2, CALM2*) were predicted to drive several downregulated ligand-receptor interactions (**Figure 4A, 4B**), mainly binding to cyclic AMP-specific phosphodiesterases (PDEs) (*PDE1A, PDE1B, PDE1C*) on immune cells in both tissues upon infection (**Supplementary Figure 8**). PDEs, whose activation is calcium/calmodulin dependent, are responsible for cyclic AMP (cAMP) degradation in T cells, which is a potent inhibitor of T-cell activation (Bjørgo et al., 2011). Hence, the downregulation of CALM-PDEs interactions following SARS-CoV-2 infection implies an increase in intracellular cAMP in T cells, and consequently an inhibition of their activity. This could represent another way during SARS-CoV-2 infection to evade the immune activation and viral clearance.

Supplementary Figures

Supplementary Figure 1

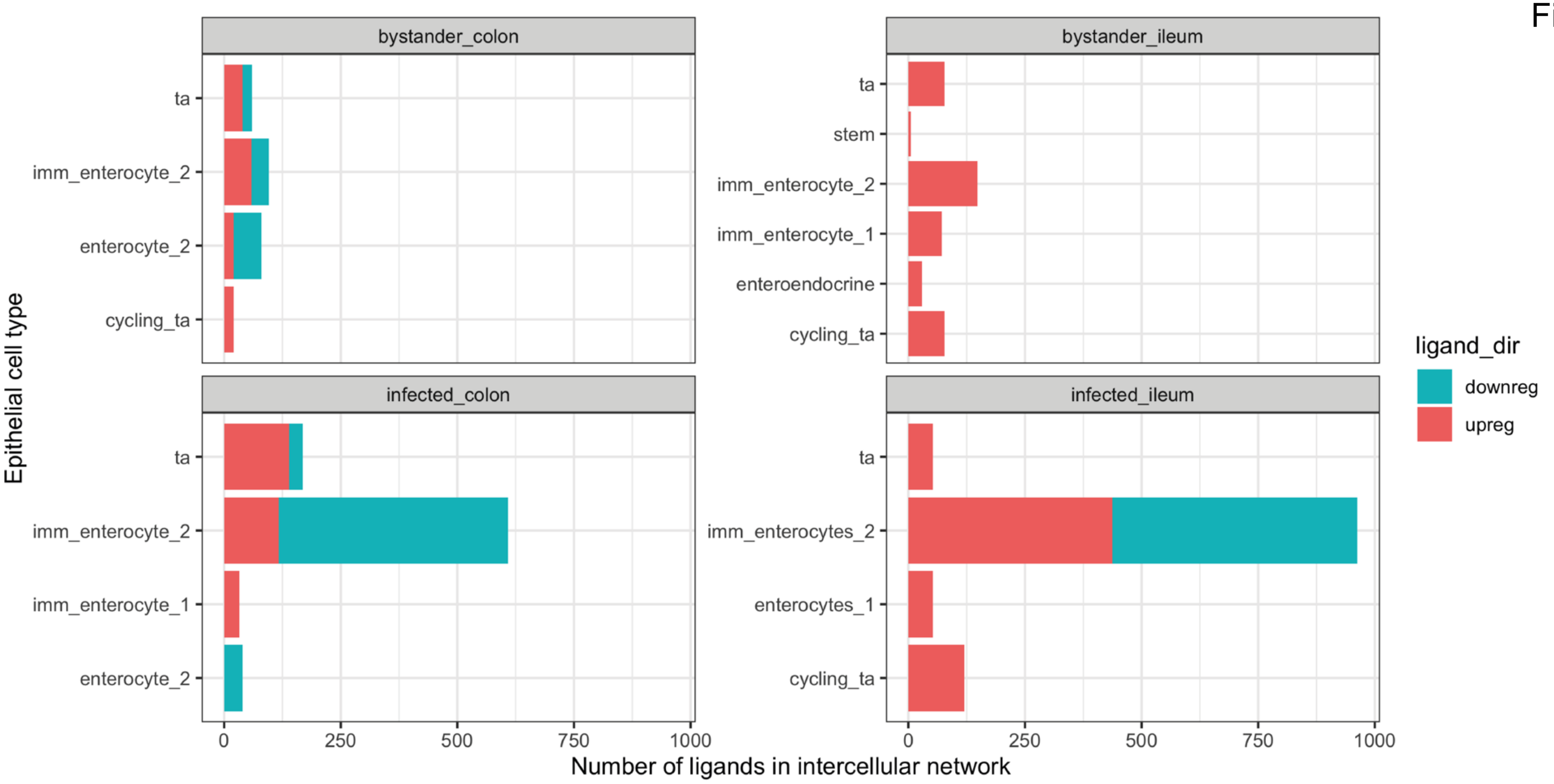

Supplementary Figure 1. Differentially expressed ligands upon SARS-CoV-2 infection in infected or bystander epithelial sub-populations. Bar chart indicating the number of differentially expressed ligands in the intercellular network in each epithelial sub-populations, either bystander or infected, in ileal or colonic organoids infected with SARS-CoV-2 vs control (24 hrs). Differentially expressed ligands are those DEGs found in (Lamers et al., 2020; Stanifer et al., 2020; Triana et al., 2021a; Zang et al., 2020), for which at least one binding receptor was found on immune cell populations. Color of the bar indicates the direction of regulation (red, upregulated; blue: downregulated). Abbreviations: ta, transit amplifying; imm_enterocyte, immature enterocyte.

Supplementary Figure 2

ViralLink

Colon

Network characteristics

Network parameter	Statistics
Number of nodes	1423
Number of edges	9971
Average number of neighbours	14
Network diameter	10
Path length	4

Node table

	Viral miRNAs or proteins	Human Binding proteins	Intermediary signalling proteins	TFs	Differentially expressed ligands	Total
Viral miRNA specific	19	107	120	4	2	**252**
Viral protein specific	28	236	223	4	2	**493**
Shared	0	66	565	29	18	**678**
**Total**	**47**	**409**	**908**	**37**	**22**	**1423**

Ileum

Network characteristics

Network parameter	Statistics
Number of nodes	1316
Number of edges	7935
Average number of neighbours	12
Network diameter	9
Path length	4

Node table

	Viral miRNAs or proteins	Human Binding proteins	Intermediary signalling proteins	TFs	Differentially expressed ligands	Total
Viral miRNA specific	28	125	242	6	1	**402**
Viral protein specific	19	214	169	2	1	**405**
Shared	0	54	399	29	26	**508**
**Total**	**47**	**393**	**810**	**37**	**28**	**1315**

CARNIVAL

Colon

Network characteristics

Network parameter	Statistics
Number of nodes	147
Number of edges	279
Average number of neighbours	3.782
Network diameter	15
Path length	6.375

Node table

	Viral miRNAs or proteins	Human Binding proteins	Intermediary signalling proteins	TFs	Differentially expressed ligands	Total
Viral miRNA specific	4	0	31	22	0	**57**
Viral protein specific	16	25	4	10	0	**55**
Shared	0	2	1	11	21	**35**
**Total**	**20**	**27**	**36**	**43**	**21**	**147**

Ileum

Network characteristics

Network parameter	Statistics
Number of nodes	274
Number of edges	479
Average number of neighbours	3.219
Network diameter	9
Path length	3.353

Node table

	Viral miRNAs or proteins	Human Binding proteins	Intermediary signalling proteins	TFs	Differentially expressed ligands	Total
Viral miRNA specific	4	0	154	1	3	**162**
Viral protein specific	17	30	15	0	1	**61**
Shared	0	1	0	25	21	**49**
**Total**	**21**	**31**	**169**	**26**	**25**	**272**

Supplementary Figure 2. Intracellular signalling networks of ileal and colonic infected immature enterocytes upon SARS-CoV-2 infection. A, B) Causal networks of SARS-CoV-2-infected colonic and ileal immature enterocytes reconstructed using ViralLink or CARNIVAL. For each network the number of interacting partners (nodes), number of interactions (edges), average number of neighbours, network diameter and characteristics path length are indicated under "Network characteristics". Within the "Node table", columns indicate the types of nodes in the different layers of the network, including SARS-CoV-2 proteins or miRNAs, human binding proteins, intermediary signalling proteins, transcription factors (TFs) and differentially expressed ligands. Where an interacting partner (human protein/gene) was found to act in multiple layers of the network, it was assigned to a layer based on the following priority: differentially expressed ligands, human binding proteins, TFs, intermediary signalling proteins. Ligands have log2 fold change ≥ |0.5| and adjusted p value ≤ 0.05. Within the "Node Table", rows indicate whether the node or edge belong to intracellular signals stemming from viral miRNA only, viral protein only or both ("shared").

Supplementary Figure 3

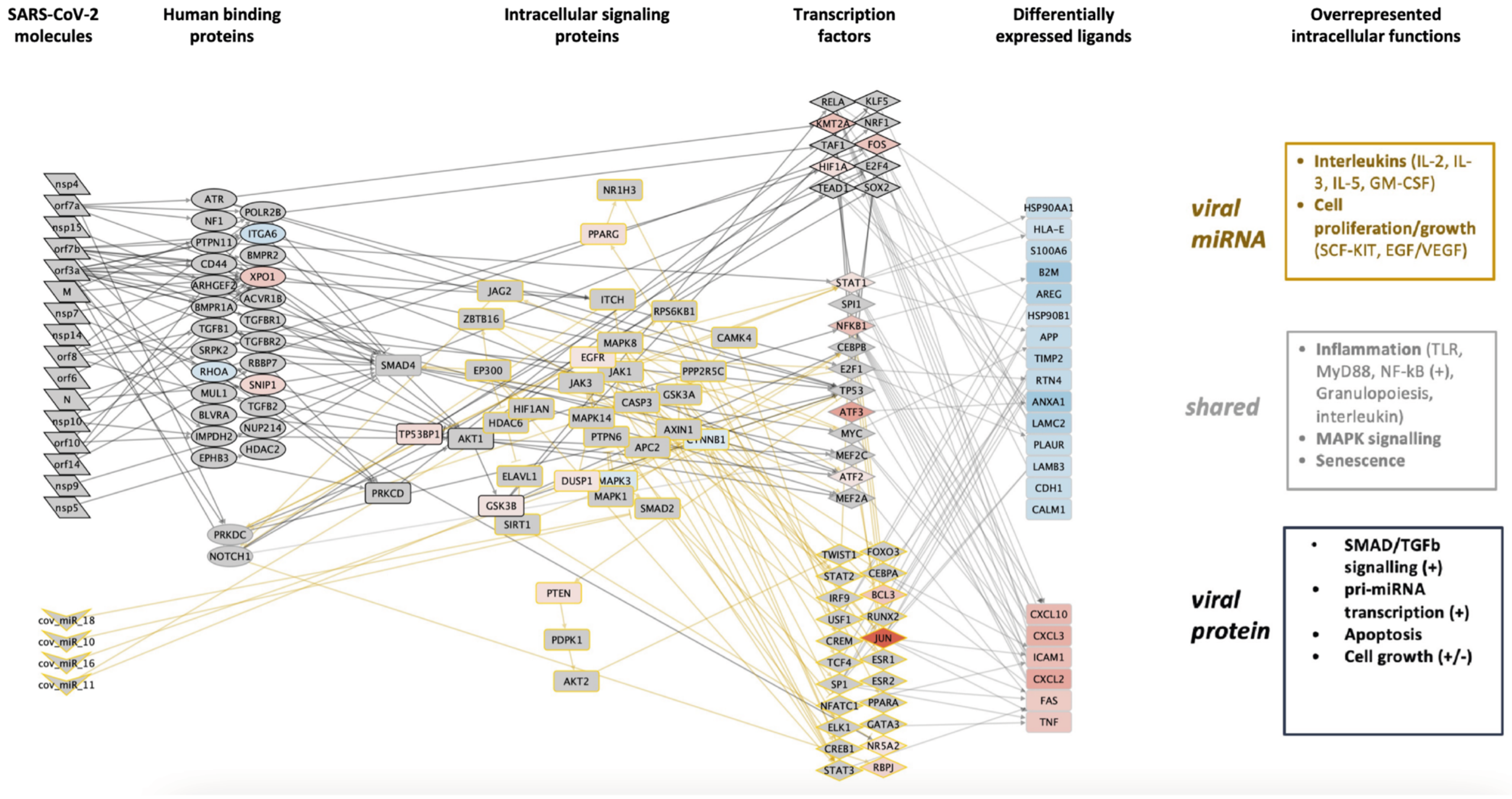

Colon

Supplementary Figure 3. **Overview of intracellular signalling upon SARS-CoV-2 infection in colonic infected immature enterocytes, reconstructed using the CARNIVAL**. From left to right: signalling cascade going from the upstream perturbation (SARS-CoV-2 proteins or miRNAs interacting with human binding proteins) to the downstream perturbation, transcription factors (TFs) regulating the differentially expressed ligands. Diamonds indicate the most active transcription factors after infection and the ovals are the perturbed human binding proteins. Rectangles are signalling intermediate proteins linking these two. Parallelograms and downward arrows indicate SARS-CoV-2 proteins and miRNAs, respectively. The color of the node indicates activation (red) or inhibition (blue) upon SARS-CoV-2 infection vs uninfected condition. Connecting edges show the direction of the interaction, as activation (pointed arrow) or inhibition (T shape arrow). Differentially expressed ligands for which no upstream signalling was identified, but downstream intercellular connections were predicted are excluded from this figure. Differentially expressed ligands are grouped based on the direction of regulation, which is indicated with blue when downregulated (bottom) and red when upregulated (top) when comparing SARS-CoV-2 infected vs uninfected conditions. Colors of the nodes edge and of the functional analysis boxes indicate if the original network was a miRNA only (yellow), viral protein only (black) or both viral protein and miRNA (grey). Functional overrepresentation analysis was carried out for the “PPI layer” of the intracellular network which includes human binding proteins, intermediary signalling proteins and TFs (adj p value < 0.05, n > 3).

Supplementary Figure 4

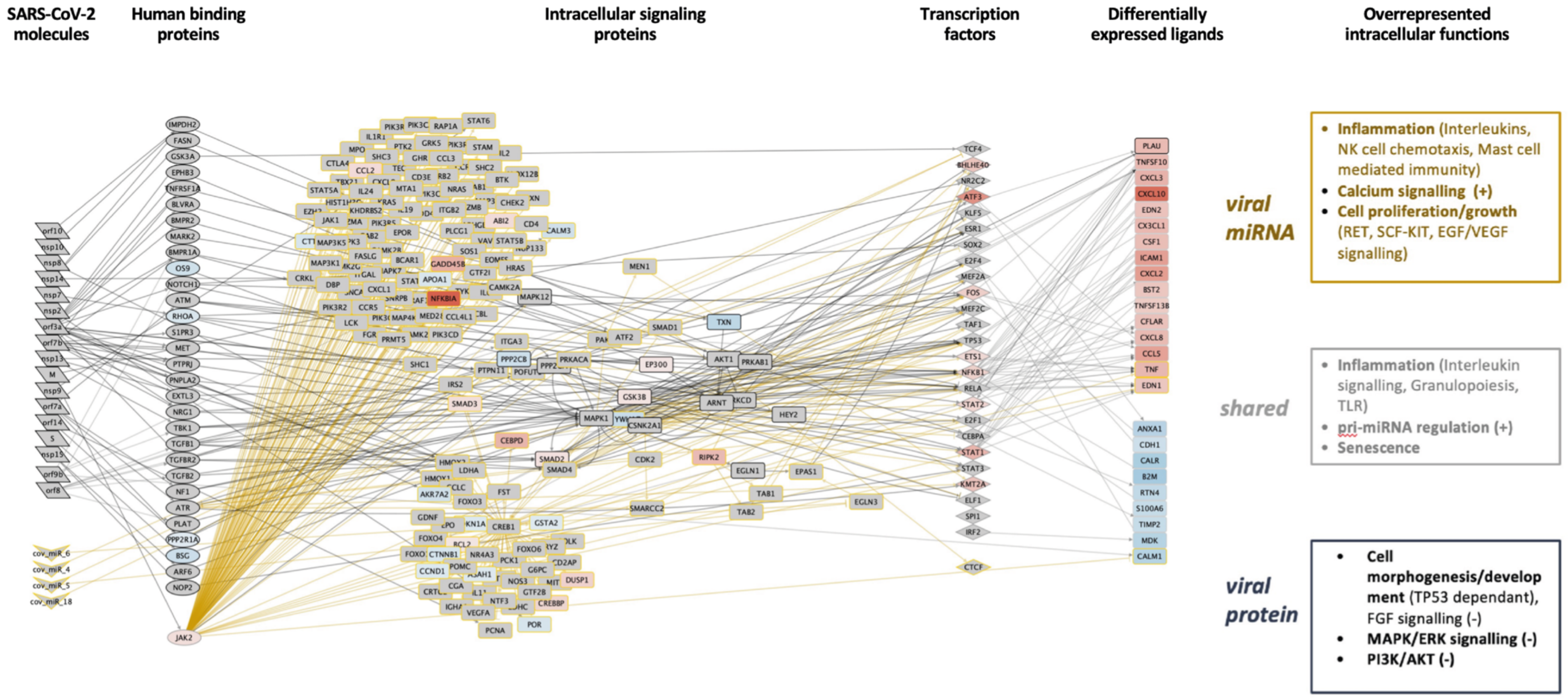

Ileum

Supplementary Figure 4. Overview of intracellular signalling upon SARS-CoV-2 infection in ileal infected immature enterocytes, reconstructed using CARNIVAL. From left to right: signalling cascade going from the upstream perturbation (SARS-CoV-2 proteins or miRNAs interacting with human binding proteins) to the downstream perturbation, transcription factors (TFs) regulating the differentially expressed ligands. Diamonds indicate the most active transcription factors after infection and the ovals are the perturbed human binding proteins. Rectangles are signalling intermediate proteins linking these two. Parallelograms and downward arrows indicate SARS-CoV-2 proteins and miRNAs, respectively. The color of the node indicates activation (red) or inhibition (blue) upon SARS-CoV-2 infection vs uninfected condition. Connecting edges show the direction of the interaction, as activation (pointed arrow) or inhibition (T shape arrow). Differentially expressed ligands for which no upstream signalling was identified, but downstream intercellular connections were predicted are excluded from this figure. Differentially expressed ligands are grouped based on the direction of regulation, which is indicated with blue when downregulated (bottom) and red when upregulated (top) when comparing SARS-CoV-2 infected vs uninfected conditions. Colors of the nodes edge and of the functional analysis boxes indicate if the original network was a miRNA only (yellow), viral protein only (black) or both viral protein and miRNA (grey). Functional overrepresentation analysis was carried out for the “PPI layer” of the intracellular network which includes human binding proteins, intermediary signalling proteins and TFs (adj p value < 0.05, n > 3).

Supplementary Figure 5

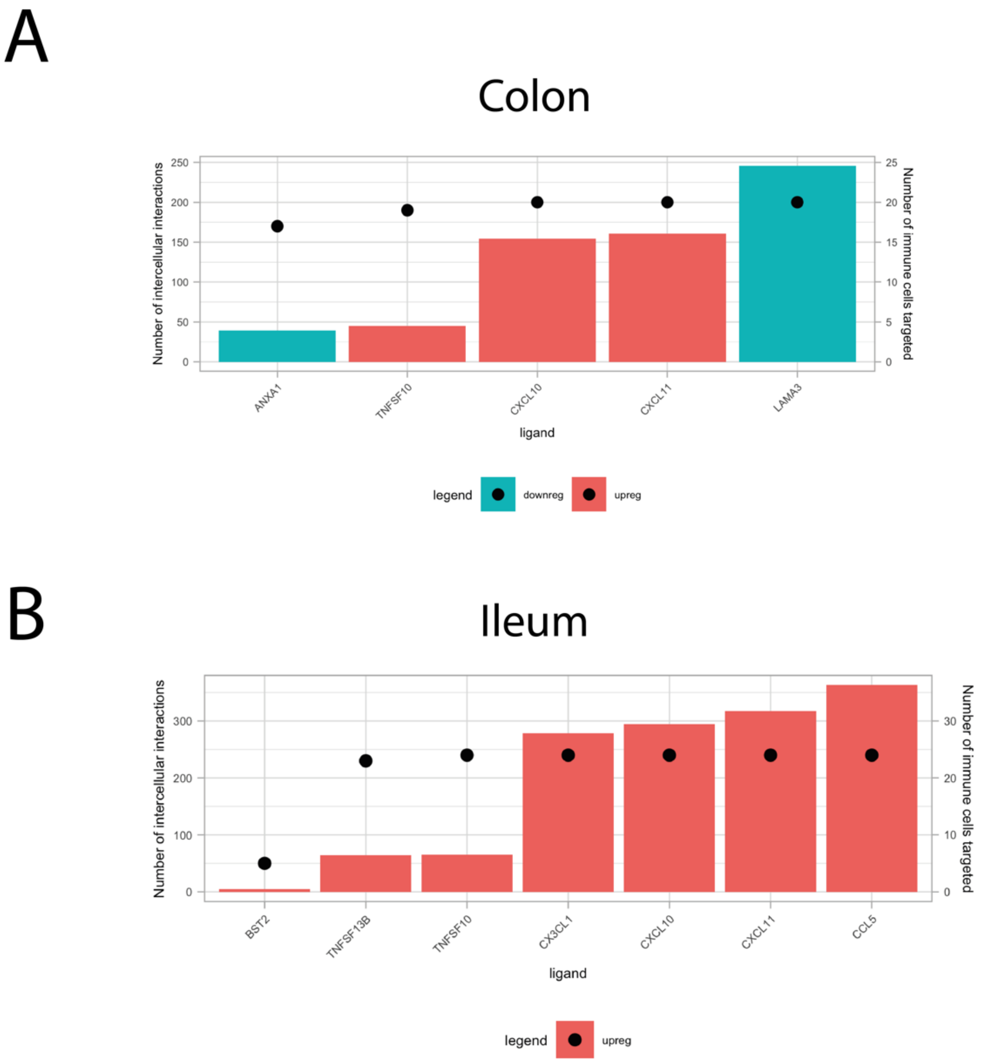

Supplementary Figure 5. Differentially expressed ligands of colonic and ileal bystander immature enterocytes upon SARS-CoV-2 infection. Bar plot showing the upregulated and downregulated ligands in the colonic (top) and ileal (bottom) bystander immature enterocytes - immune cell network scored by number of interactions (height of the bar plot) and number of immune cells targeted (black dots). Upregulated ligands are shown in red and downregulated ligands in blue.

Supplementary Figure 6

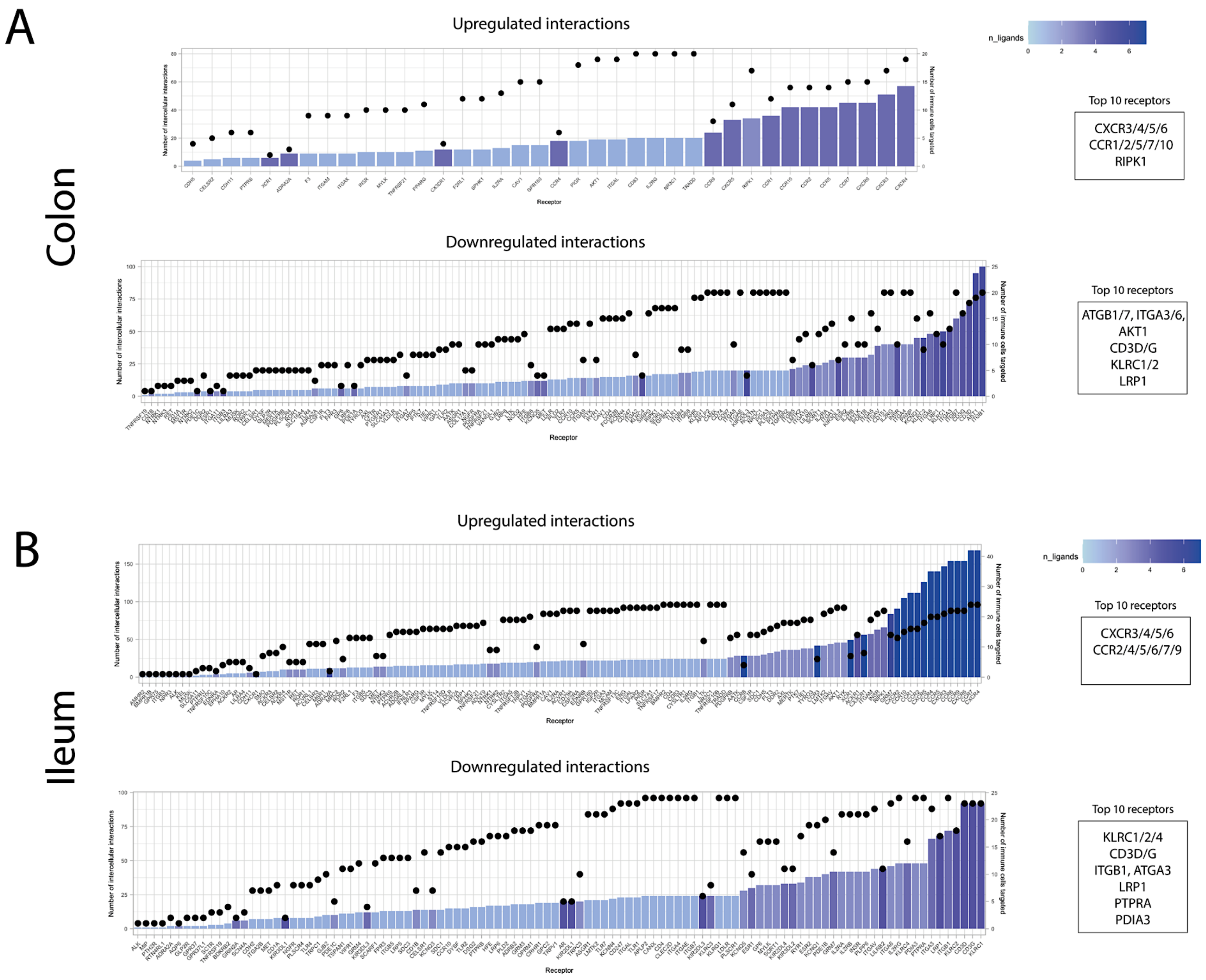

Supplementary Figure 6. Receptors involved in intercellular interactions between colonic and ileal infected immature enterocytes and resident immune cells. Bar plot showing the immune receptors targeted by upregulated (top graph) and downregulated (bottom graph) ligands in colonic (A) and ileal (B) infected immature enterocytes, scored by number of interactions (height of the bar plot) and number of immune cells targeted (black dots). The color of the bar plots indicates the number of ligands targeting each of the receptors indicated.

A

Supplementary Figure 7

Colon

Upregulated interactions

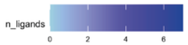

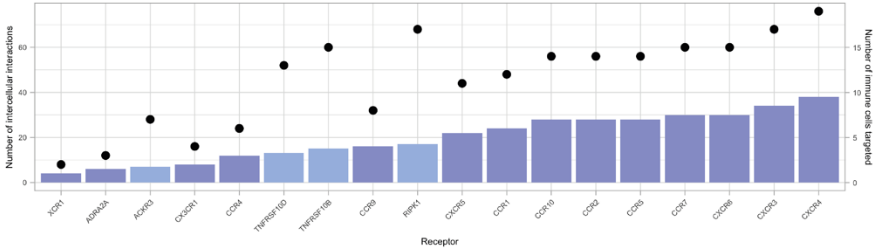

Top 10 receptors

CXCR3/4/5/6
CCR1/2/5/7/10
RIPK1

Downregulated interactions

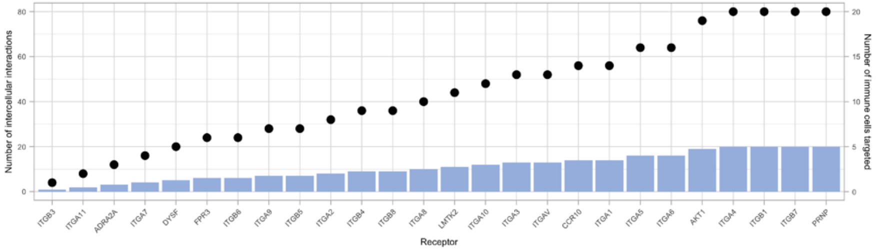

Top 10 receptors

PRNP
ITGA3/4/5/6/V
ITGB1/7
AKT1
CCR10

B

Ileum

Upregulated interactions

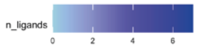

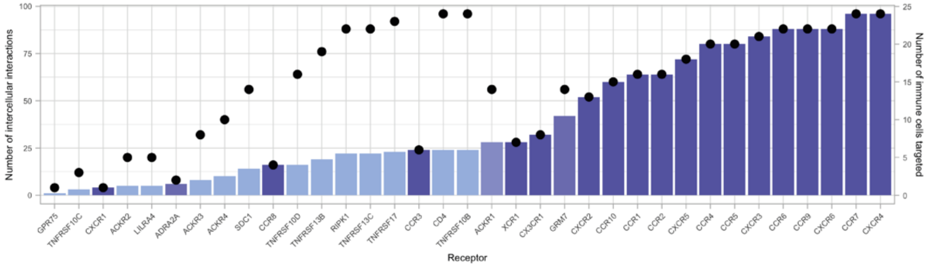

Top 10 receptors

CXCR3/4/5/6
CCR1/2/5/7/10
RIPK1

Bystander immature enterocytes

Supplementary Figure 7. Receptors involved in intercellular interactions between colonic and ileal bystander immature enterocytes and resident immune cells. Bar plot showing the immune receptors targeted by upregulated (top graph) and downregulated (bottom graph) ligands in colonic (A) and ileal (B) bystander immature enterocytes, scored by number of interactions (height of the bar plot) and number of immune cells targeted (black dots). The color of the bar plots indicates the number of ligands targeting each of the receptors indicated.

Supplementary Figure 8

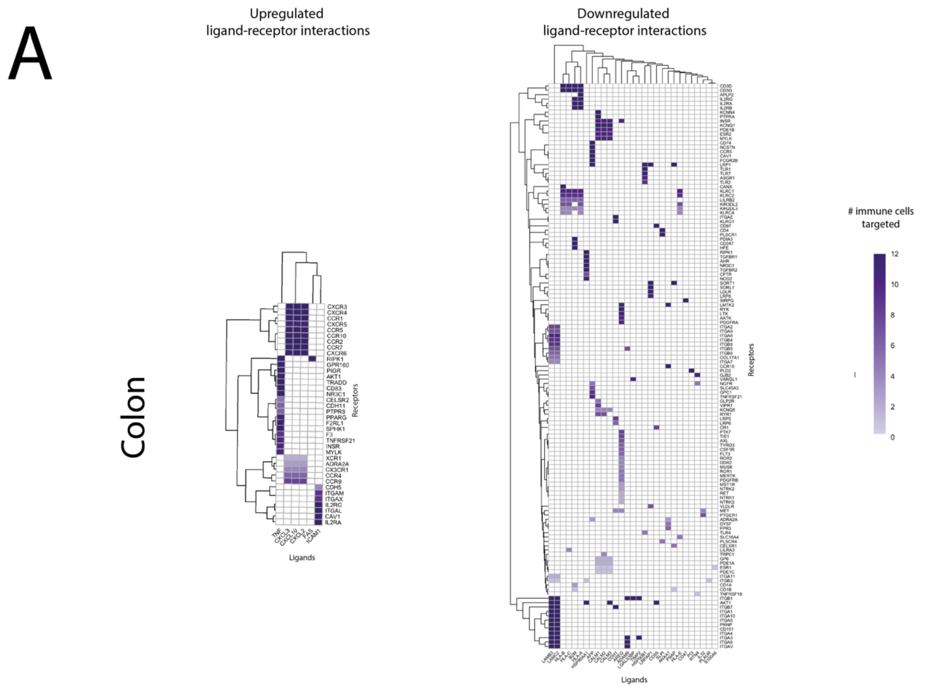

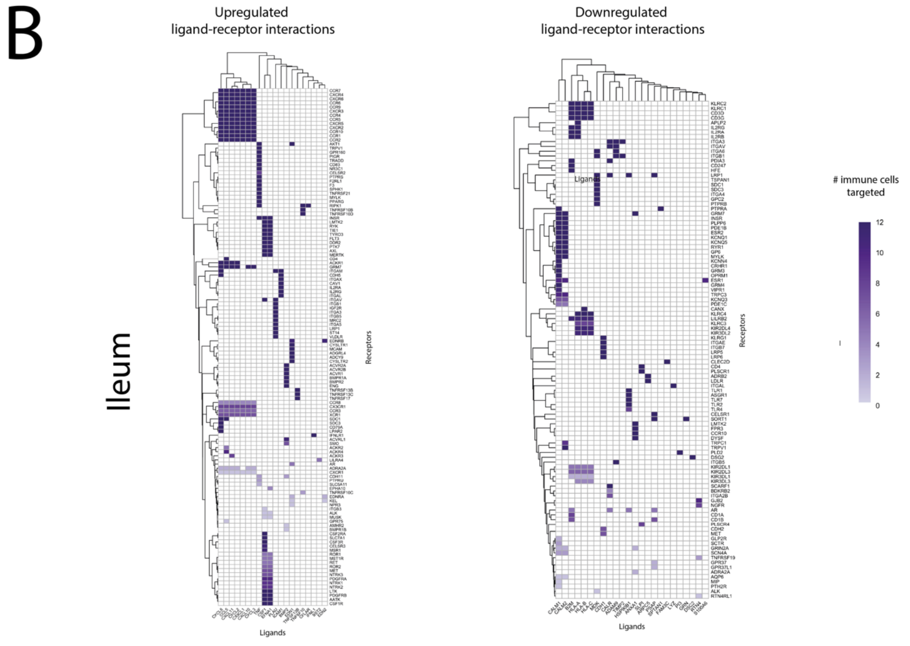

Supplementary Figure 8. Intercellular interactions with upregulated and downregulated ligands of colonic and ileal infected immature enterocytes.

A,B) Interactions driven by upregulated and downregulated ligands in the colon (A) and ileum (B) are shown separately. The number of immune cells involved in ligand-receptor interaction pair is indicated in purple.

Supplementary Figure 9

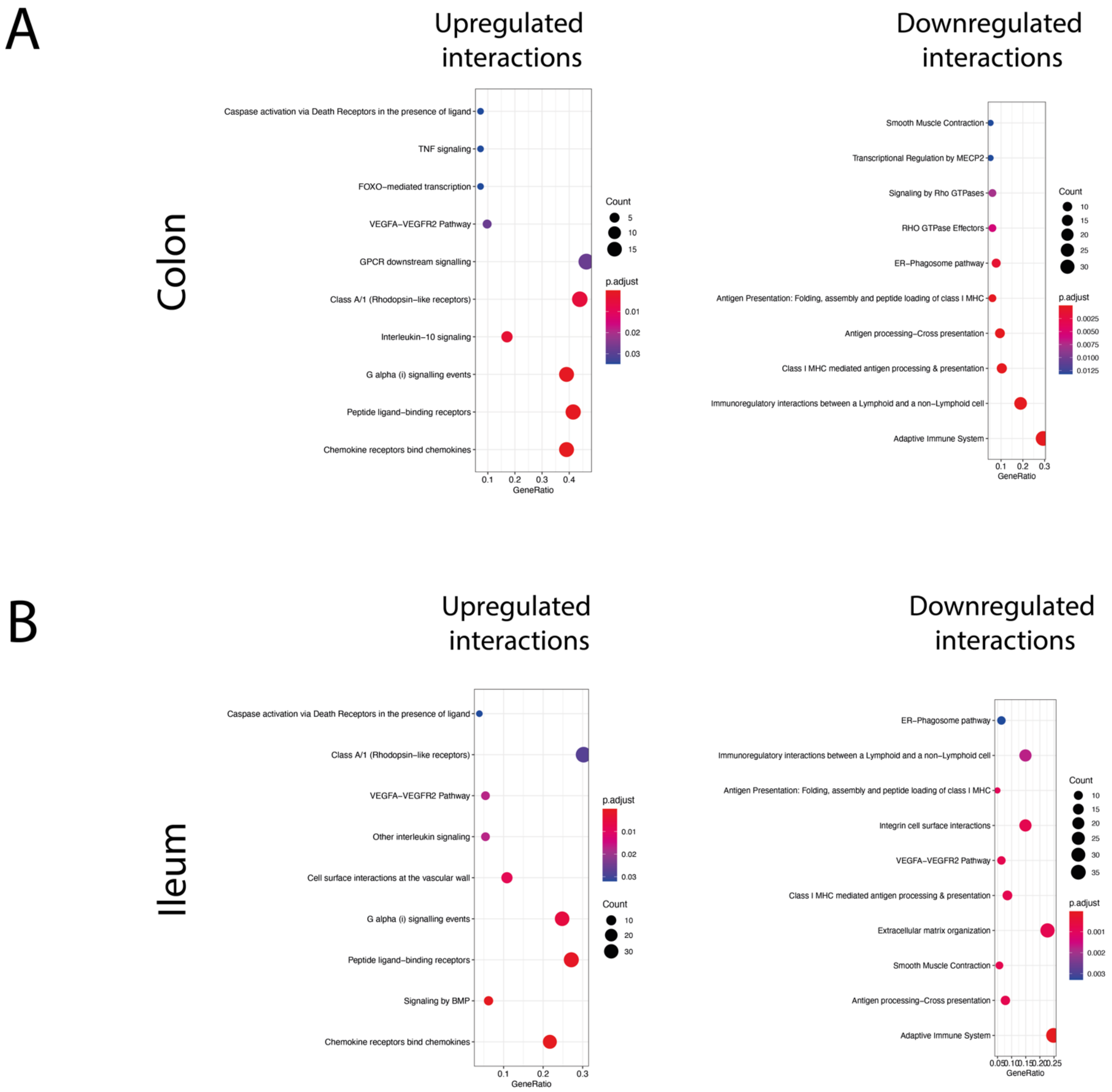

Supplementary Figure 9. Functional analysis of ligand-receptor interactions between ileal and colonic immature enterocytes and resident immune cells upon SARS-CoV-2. A, B) Reactome functional overrepresentation analysis carried out a list of all upregulated ligands and receptors for interactions of a specific condition. There was no weighting for the number of interactions of each ligand/receptor. Analyses relative to interactions driven by upregulated and downregulated ligands are shown separately.

Supplementary Figure 10

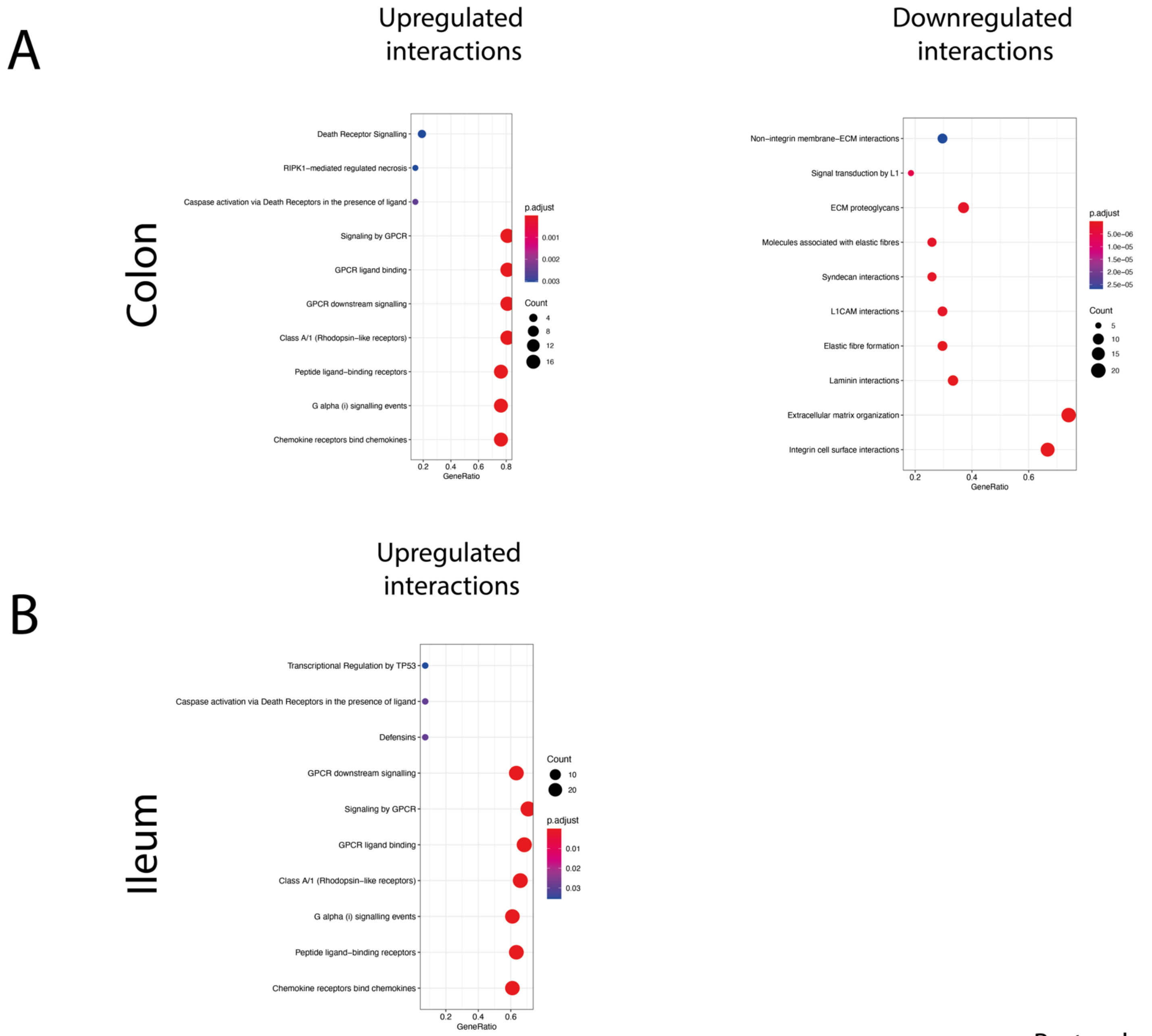

Bystander immature enterocytes

Supplementary Figure 10. Functional analysis of ligand-receptor interactions between ileal and colonic immature enterocytes and resident immune cells upon SARS-CoV-2. A, B) Reactome functional overrepresentation analysis carried out a list of all upregulated ligands and receptors for interactions of a specific condition. There was no weighting for the number of interactions of each ligand/receptor. For the colon (A), analyses relative to interactions driven by upregulated and downregulated ligands are shown separately. For the ileum (B), analyses relative to upregulated ligands only are shown, as there were no interactions driven by downregulated ligands.

Supplementary Figure 11

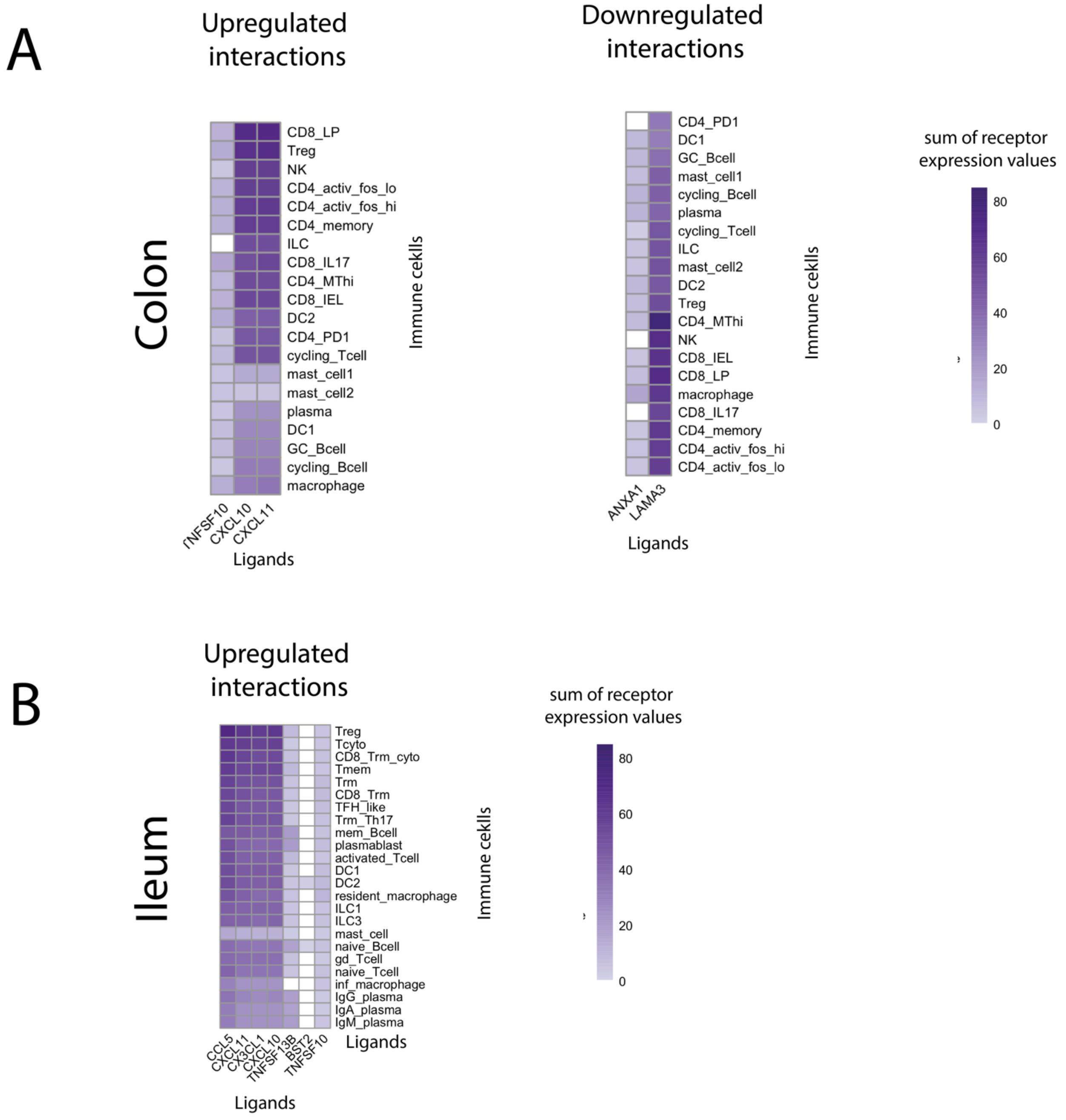

Bystander immature enterocytes

Supplementary Figure 11. Ligands of bystander immature enterocytes involved in the strongest up and downregulated interactions upon SARS-CoV-2 infection in the colon and ileum. (A, B) Heatmap showing the upregulated and downregulated interactions in the colon (A) and ileum (B) between intestinal epithelial ligands and resident immune cells of bystander immature enterocytes upon infection. The strength of the interaction is expressed by accounting for the number of interactions between epithelial ligands and immune receptors and the level of receptor expression of immune cells. The strength of the interaction, named "sum of expression values", is visualized using a color gradient from white (weakest interactions) to purple (strongest interactions). Abbreviations: Ileum: CD8_Trm_cyto, Resident memory cytotoxic T cell; DC2, dendritic cell 2; Trm, Tissue-resident memory T cell, gd_Tcell, Gamma delta (γδ) T cells; ILC, Innate lymphoid cell; mem_Bcell, memory B cell; naive_Bcell, naive B cell; TFH_like, T follicular helper cells; Trm_Th17, Tissue-resident memory Th17 cells; Treg, Regulatory T cell; Tcyto, Cytotoxic T cell; Tmem, Memory T cells. Colon: ILC, Innate lymphoid cell; CD8_IL17, IL-17+ CD8+ T cells; DC, dendritic cells; GC_Bcell, Germinal center B cells; CD4_PD1, mast, mast cell; Treg, Regulatory T cell; NK, Natural Killer cell, CD4_MThi, high mitochondrial CD4+ T cell; CD4_memory, CD4+ Memory T cell, CD4_activ_fos_high, activated CD4+ T cells (high/low c-fos); CD8_LP, CD8+ lymphocyte-predominant cells, CD8_IEL, CD8+ intraepithelial lymphocytes.

Supplementary Figure 12

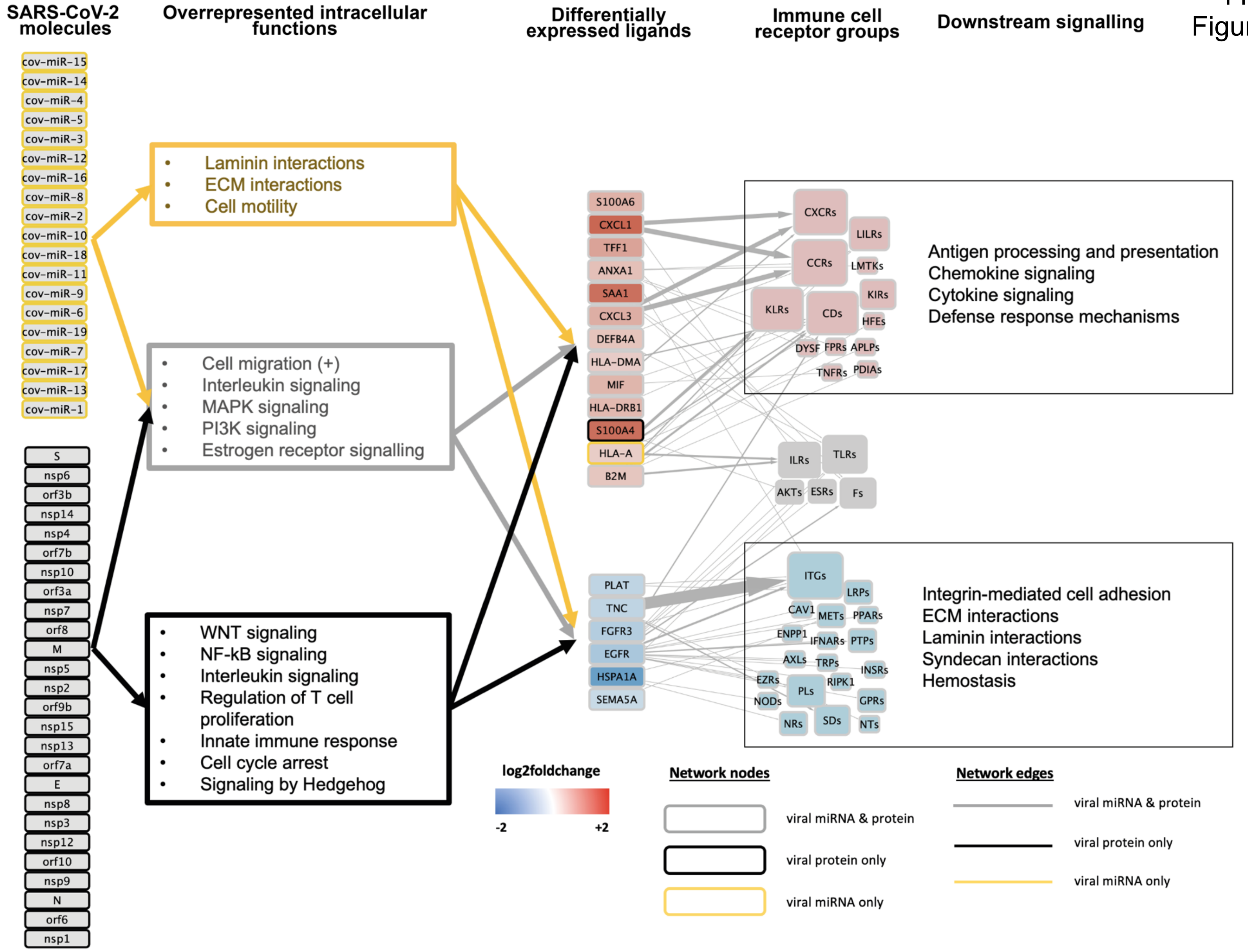

Supplementary Figure 12. Overview of intracellular and intercellular signalling of upper airway ciliated epithelial cells of moderate COVID-19 patients. From left to right: signalling cascade going from SARS-CoV-2 molecules (proteins or miRNAs) to differentially expressed ligands on ciliated epithelial cells and binding receptor groups on immune cells. Intracellular network: SARS-COV-2 molecules are grouped separately if they are viral proteins (bottom) or miRNAs (top). Differentially expressed ligands for which no upstream signalling was identified, but downstream intercellular connections were predicted are excluded from this figure. Differentially expressed ligands are grouped based on the direction of regulation, which is indicated with blue when downregulated (bottom) and red when upregulated (top) when comparing SARS-CoV-2 infected vs uninfected conditions. Colors of the nodes and of the functional analysis indicate if the original network was a miRNA only (yellow), viral protein only (black) or both viral protein and miRNA (grey). Functional overrepresentation analysis was carried out for the "PPI layer" of the intracellular network which includes human binding proteins, intermediary signalling proteins and TFs (adj p value < 0.05, $n > 3$). Intercellular network: Size of the receptor node represents the sum of receptors within the group targeted by each incoming ligand. Functional analysis is indicated for ligand-receptor groups. Receptor groups layout is based on whether they contributed to the functional analysis of upregulated interactions (red) or downregulated interactions (blue). Receptor groups not contributing to any functions are indicated in light grey.

Supplementary Figure 13

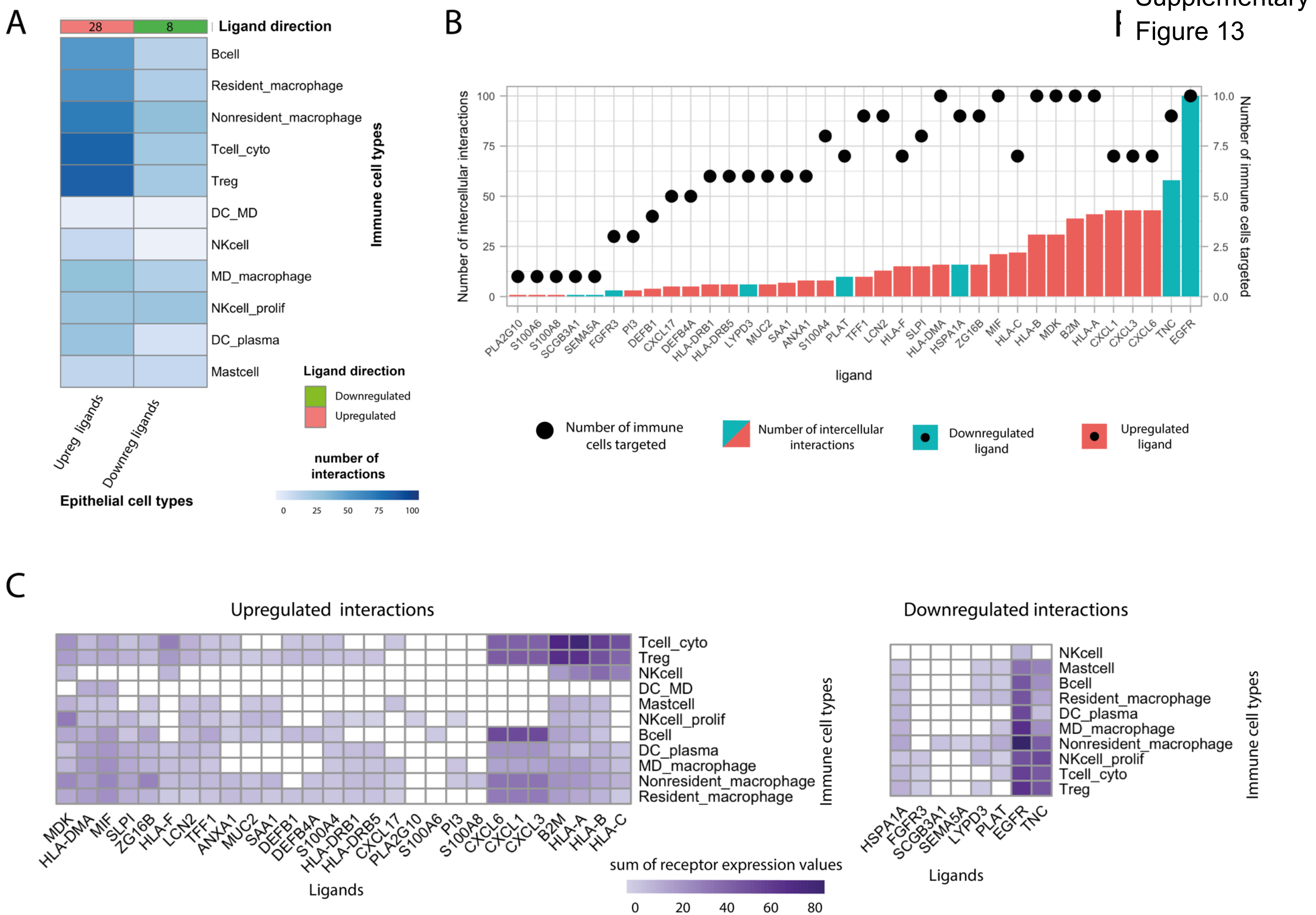

Supplementary Figure 13. **Analysis of the intracellular and intercellular networks of upper airways ciliated epithelial cells of moderate COVID-19 patients. (A)** Heatmap showing the number of intercellular interactions between ciliated epithelial cells and resident immune cells. Interactions driven by upregulated and downregulated ligands (ligand direction) are shown separately. The intensity of the color indicates the number of interactions with the immune cell types whose receptor is targeted by the epithelial cells ligands. The numbers on the ligand direction row refer to the number of upregulated or downregulated ligands driving the indicated interactions with immune cells for the different groups/conditions. Abbreviations: Tcell_cyto, cytotoxic T cell; DC_MD, MD Dendritic cell; NKcell_prolif, proliferating NKT cell; DC_plasma, Plasmacytoid dendritic cell; Treg, Regulatory T cell.

(B) Bar plot showing the upregulated and downregulated ligands in ciliated epithelial cells - immune cell network scored by number of interactions (height of the bar plot) and number of immune cells targeted (black dots). Upregulated ligands are shown in red and downregulated ligands in blue. **(C)** Heatmap showing the upregulated and downregulated interactions between ciliated epithelial cell ligands and resident immune cells in moderate COVID-19. The strength of the interaction is expressed by accounting for the number of interactions between epithelial ligands and immune receptors and the level of receptor expression of immune cells. The strength of the interaction, named “sum of expression values”, is visualized using a color gradient from white (wekeast interactions) to purple (strongest interactions). Abbreviations: *Abbreviations:* Tcell_cyto, cytotoxic T cell; DC_MD, MD Dendritic cell; NKcell_prolif, proliferating NKT cell; DC_plasma, Plasmacytoid dendritic cell; Treg, Regulatory T cell.
